# Histone H3 lysine 4 methylation signature associated with human undernutrition

**DOI:** 10.1073/pnas.1722125115

**Published:** 2018-11-12

**Authors:** Robin Uchiyama, Kristyna Kupkova, Savera J. Shetty, Alicia S. Linford, Marilyn G. Pray-Grant, Lisa E. Wagar, Mark M. Davis, Rashidul Haque, Alban Gaultier, Marty W. Mayo, Patrick A. Grant, William A. Petri, Stefan Bekiranov, David T. Auble

**Affiliations:** ^a^Division of Infectious Diseases and International Health, University of Virginia Health System, Charlottesville, VA 22908;; ^b^Department of Biochemistry and Molecular Genetics, University of Virginia Health System, Charlottesville, VA 22908;; ^c^Department of Biomedical Engineering, Brno University of Technology, 61200 Brno, Czech Republic;; ^d^Department of Microbiology and Immunology, Stanford University, Stanford, CA 94305;; ^e^Howard Hughes Medical Institute, Stanford University, Stanford, CA 94305;; ^f^Laboratory Sciences Division, International Centre for Diarrhoeal Disease Research, Dhaka 1000, Bangladesh;; ^g^Department of Neuroscience, Center for Brain Immunology and Glia, University of Virginia Health System, Charlottesville, VA 22908

**Keywords:** epigenetics, undernutrition, histone methylation

## Abstract

Chronically undernourished children become stunted during their first 2 years and thereafter bear burdens of ill health for the rest of their lives. Contributors to stunting include poor nutrition and exposure to pathogens, and parental history may also play a role. However, the epigenetic impact of a poor environment on young children is largely unknown. Here we show the unfolding pattern of histone H3 lysine 4 trimethylation (H3K4me3) in children and mothers living in an urban slum in Dhaka, Bangladesh. A pattern of chromatin modification in blood cells of stunted children emerges over time and involves a global decrease in methylation at canonical locations near gene start sites and increased methylation at ectopic sites throughout the genome. This redistribution occurs at metabolic and immune genes and was specific for H3K4me3, as it was not observed for histone H3 lysine 27 acetylation in the same samples. Methylation changes in stunting globally resemble changes that occur in vitro in response to altered methylation capacity, suggesting that reduced levels of one-carbon nutrients in the diet play a key role in stunting in this population. A network of differentially expressed genes in stunted children reveals effects on chromatin modification machinery, including turnover of H3K4me3, as well as posttranscriptional gene regulation affecting immune response pathways and lipid metabolism. Consistent with these changes, reduced expression of the endocytic receptor gene LDL receptor 1 (LRP1) is a driver of stunting in a mouse model, suggesting a target for intervention.

## Significance

The early life environment can exert a profound effect on long-term health. However, differences in developmental epigenetic patterns in response to environmental challenges are not well understood in humans, where nutrient insufficiency and pathogen exposure in early infancy can impact immune system function and metabolic health into adulthood. Here we report a comprehensive global picture of the patterns of the epigenetic modification histone H3 lysine 4 trimethylation (H3K4me3) in undernourished infants and their mothers. Comparisons of the emergent patterns of H3K4me3 within the first year of life reveal large-scale changes consistent with the impact of a poor environment, and uncovered a candidate gene with a role in the response, which was validated in a mouse model.

**Fig. 1.**
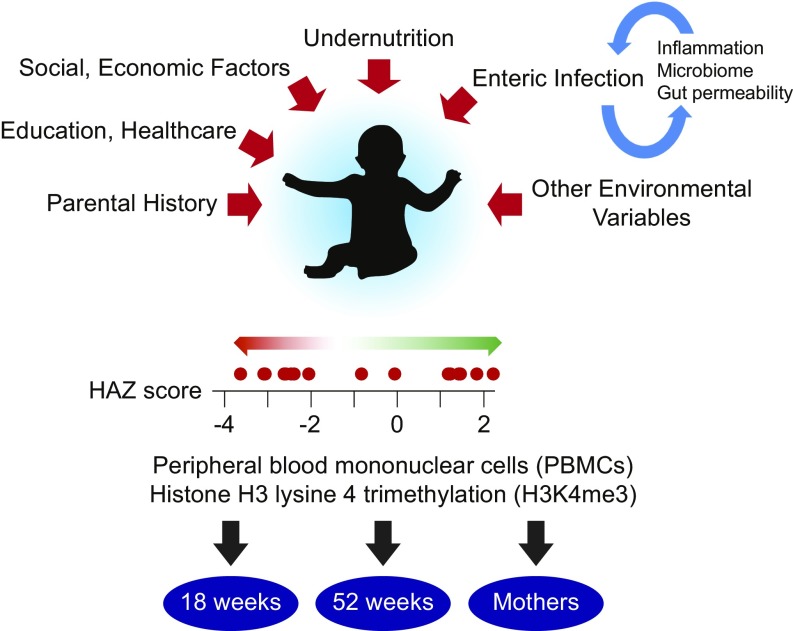
Outline of the problem and experimental approach. Factors contributing to childhood undernutrition are marked with red arrows. Enteric infection can lead to gut dysfunction that makes reinfection more likely, a condition known as environmental enteropathy. Undernourished children are stunted, which is defined by a HAZ score < −2. The red dots on the HAZ scale correspond to scores of children at 1 y of age whose data were analyzed. Genome-wide maps of H3K4me3 were obtained using PBMCs from children at 18 wk and 52 wk of age, as well as their mothers.

Nutrient insufficiency contributes to poor health in one in four children worldwide (1). Additionally, undernutrition is responsible for up to one-third of the deaths of children under 5 y of age (2). Undernourished children are growth-impaired (stunted), suffer from vaccine failure and cognitive impairment, and health effects that occur within the first ∼2 y of life can persist even if nutrition becomes adequate later in life, potentially even impacting the health and behavior of subsequent offspring who never directly experienced nutrition limitation (3–7). In addition to food insecurity, undernutrition is likely attributable to many environmental factors (Fig. 1), including chronic cycles of infection by pathogens found in areas without reliable clean water, causing bouts of diarrhea that deplete children of essential nutrients, compromise intestinal barrier function, and cause inflammation (1, 8). As a consequence, children in both food-secure and food-insecure households in the developing world are at risk to be undernourished (1). Additional determinants of undernutrition at 1 y of age include being undernourished at birth and having a short-statured mother (9). These results are consistent with an epigenetic and possibly transgenerational contribution to the development of stunting. The evidence indicates that immune cells play a fundamentally important role in the pathogenesis of stunting. Immune cells are highly active sensors of overall metabolic health, and healthy metabolism is central for mounting proper immune responses (10, 11). In addition, immune cells are key players in the physiological responses to general nutrient availability, as well as fighting infection (10, 12). Conversely, immune cell dysfunction has been functionally linked to malnutrition (13–16), reinforcing the importance of understanding immune cell status in the context of stunting. It has been estimated that the use of all known effective interventions in 99% of children would only decrease stunting by about one-third (2). For this reason, there is a pressing need to better understand the events that unfold on a molecular level in infants and give rise to the stunted phenotype, and to identify new targets and strategies for intervention.

## Results

### Global Patterns of H3K4me3 in Stunted and Control Children.

To determine whether undernourished children have a distinct epigenetic signature, we combined chromatin immunoprecipitation with high-throughput DNA sequencing (ChIP-seq) to inventory the distribution and abundance of histone H3 lysine 4 trimethylation (H3K4me3) in peripheral blood mononuclear cell (PBMC) samples from children and their mothers enrolled in the PROVIDE (“performance of rotavirus and oral polio vaccines in developing countries”) study birth cohort in Dhaka, Bangladesh (17) (Fig. 1 and *SI Appendix*, *Experimental Methods and Materials* and Table S1). H3K4me3 was chosen because of its notable association with transcription start sites (TSSs) and because the extent of modification at the TSS is correlated with gene activity (18). The PROVIDE study is an on-going longitudinal study that enrolled ∼700 children within the first week of life in an urban Dhaka slum. Stunting emerges within the first 2 y of life and is defined by a height-for-age *z*-score (HAZ score) <−2 at 1 y of age. Nutritional status is followed by anthropometry at every study visit, with undernutrition (as measured by stunting) increasing from 9.5% of the population at birth to 27.6% at 12 mo of life (19).

**Fig. 2.**
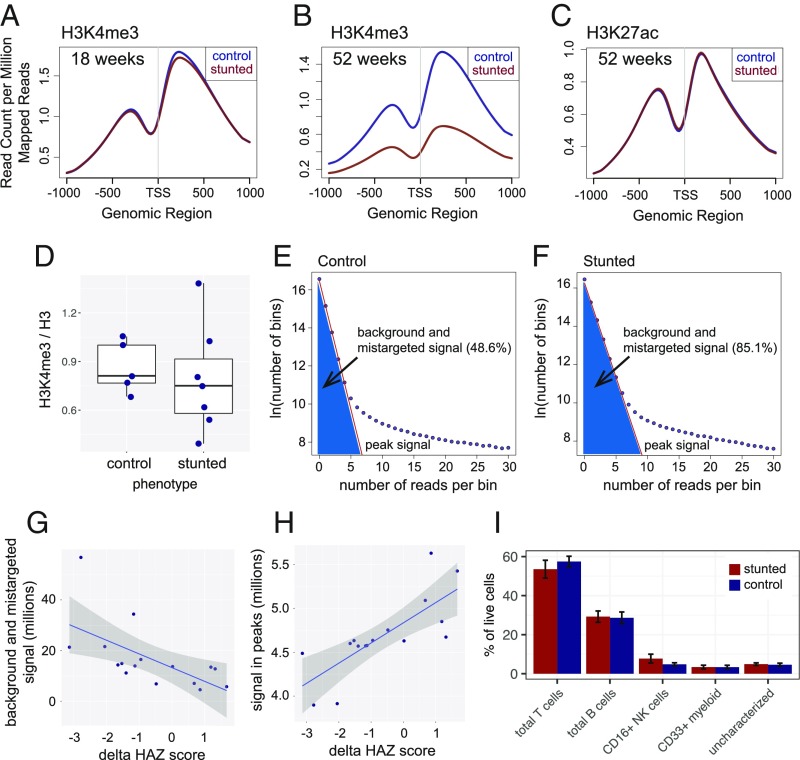
Global change in H3K4me3 in control and stunted children at 1 y of age. (*A* and *B*) Gene average plots of histone H3K4me3 levels with respect to TSS at 18 and 52 wk of age in control and stunted children. Stunted children were phenotypically defined by HAZ score < −2 at 1 y of age. The TSS methylation difference at 1 y of age is highly significant (*P* < 2.2e-16 by Wilcoxon rank sum test). (*C*) Gene average plot of H3K27ac levels in control and stunted children at 52 wk of age. (*D*) Total histone H3K4me3 relative to total histone H3 in 1-y-old control and stunted children determined by Western blotting. Each dot represents the H3K4me3/H3 ratio in an individual child’s PBMC chromatin sample. (*E* and *F*) Representative read distributions across the genome. The plots show the number of reads per 150-bp bin (*x* axis) versus the logarithm of the number of bins (*y* axis) with the indicated read count for a dataset from a control child and a dataset from a stunted child, both at 1 y of age. Background plus mistargeted read counts were calculated from the linear fit of bins using an exponential distribution background model (as detailed in *Methods*) with the lowest read counts (red lines; typically zero to nine reads per bin, optimized for each dataset). (*G*) Relationship between background plus mistargeted signal (calculated as in *E* and *F*) and ∆HAZ score. (*H*) Relationship between H3K4me3 signal in peaks and ∆HAZ score. In *G* and *H*, linear fits of the data are shown in blue with SE indicated by shading. (*I*) PBMC cell subpopulations in control and stunted children from age 53 wk. Percentages were calculated by manual gating of flow cytometry data. Data are a summary of 8 stunted and 11 nonstunted children. Bar positions represent the mean and error bars indicate SE.

H3K4me3 signal was notably enriched proximal to TSSs as expected, and when normalized to the total number of mapped reads, this enrichment was indistinguishable in 18-wk control children and children of the same age who became stunted by 1 y (Fig. 2*A* and *SI Appendix*, Fig. S1*A*). An extensive analysis of the data from the 18-wk-old children revealed little to no significant H3K4me3 changes associated with either their HAZ score or a number of 18-wk biomarkers acquired in the PROVIDE study (19). In contrast, TSS-proximal H3K4me3 was notably decreased in stunted children at 1 y of age (Fig. 2*B*). Importantly, analysis of histone H3 K27 acetylation (H3K27ac) showed no such global shift with stunting (Fig. 2*C*), despite the modification being localized to similar sites as H3K4me3 genome-wide (*SI Appendix*, Fig. S1 *B*–*D*) (20). H3K27ac data were acquired using the same samples as were used for H3K4me3 ChIP-seq, indicating that the global effect on chromatin modification in stunting is highly specific for H3K4me3. In addition, Western blot analysis revealed no significant correlation between the total H3K4me3 level relative to total histone H3 and the stunted phenotype (Fig. 2*D* and *SI Appendix*, Fig. S1*E*), nor was there any correlation between total H3K4me3 and an individual’s HAZ score. These observations suggest that the average decrease in H3K4me3 at the TSS in stunted 1-y-old children was not due to a global decrease in total H3K4me3, but rather a redistribution of H3K4me3 from TSSs to other regions in the genome. Indeed, the read count distribution in 150-bp bins tiled across the genome was consistent with such a broad but low-level increase in H3K4me3 signal at non-TSS regions throughout the genome in stunted children (Fig. 2 *E* and *F*).

Importantly, while an exponential read-count distribution of H3K4me3 has not been reported before, this model described the ENCODE H3K4me3 read distribution as well (*SI Appendix*, Fig. S2*A*), suggesting a previously unrecognized general feature of the distribution of H3K4me3 genome-wide. Despite the reduced average peak height in samples from stunted children, sets of significant H3K4me3 peaks identified separately in control and stunted datasets were broadly overlapping (*SI Appendix*, Fig. S2*B*), indicating that the more dispersed nature of H3K4me3 in stunted individuals did not significantly impact our ability to detect peaks of enrichment. To better understand the relationship between the landscape of H3K4me3 and a child’s growth within the first year, we investigated the relationship between H3K4me3 signal and ΔHAZ, a measure of growth trajectory, which we define as the change in HAZ score from birth to 1 y of age. Consistent with the decreased average signal at TSSs in 1-y-old stunted children, the H3K4me3 signal in peaks was correlated with ΔHAZ score, and there was a compensatory increase in the H3K4me3 signal in nonpeak regions in children with lower compared with higher ΔHAZ scores (Fig. 2 *G* and *H*). To further substantiate the conclusion that H3K4me3 signal was globally redistributed in stunted compared with control children, we obtained and analyzed post hoc four H3K4me3 datasets from 1-y-olds using libraries prepared using *Drosophila* spike-in chromatin (*SI Appendix*, Table S2). Spike-in normalization yielded a set of differentially affected H3K4me3 peaks that broadly overlapped with those obtained by “standard” DESeq2 normalization, which assumes no significant change in total signal across samples (*SI Appendix*, Figs. S2 *C*–*F* and S3 *A* and *B*).

Importantly, there were no significant differences in the frequencies of major constituent PBMC cell subtypes in control versus stunted children at 1 y of age (Fig. 2*I*), indicating that the large-scale H3K4me3 pattern differences seen in control and stunted children were not attributable to changes in the proportions of these cells in the children’s blood. Taken together, these results support a model in which stunting is associated with the large-scale redistribution of H3K4me3 away from TSS-proximal regions and to numerous ectopic sites. Additional support for this conclusion is described below.

### Genes and Regulatory Regions Associated with Methylation Changes.

**Fig. 3.**
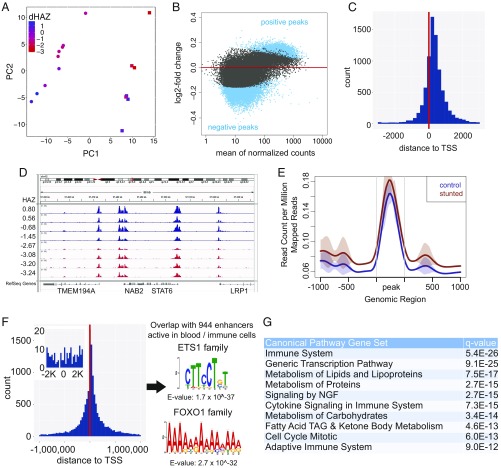
Differential H3K4me3 signature correlated with ΔHAZ (growth trajectory) at 1 y of age. (*A*) PC analysis plot of H3K4me3 datasets from 1-y-old children (with 47% and 19.8% of the variance in the data explained by PC1 and PC2, respectively). Each dot represents a child’s dataset. Data point color corresponds to the child’s ∆HAZ score, as indicated on the scale bar. Circles indicate girls, squares indicate boys. (*B*) Normalized mean H3K4me3 signal is plotted versus the log_2_ fold-change per unit of ∆HAZ score in data from 1-y-old children. Each dot represents a peak; blue dots have FDR-corrected *P* < 0.05. (*C*) Distribution with respect to the TSS of H3K4me3 peaks positively correlated with ΔHAZ score. Approximately 62% of the significantly affected peaks are within 2 kb of the TSS. (*D*) Genome browser screenshot showing H3K4me3 read distributions (normalized to total read count) in four control (blue) and four stunted (red) individuals at 1 y of age. Datasets were selected to represent the range in HAZ scores, as indicated. Note that peaks in stunted children tend to be smaller than peaks in control children. (*E*) Average plots of histone H3K4me3 levels at peaks negatively correlated with ΔHAZ score in control and stunted children. Stunted children were phenotypically defined, as in Fig. 2*A*. (*F*) Distribution with respect to the TSS of H3K4me3 peaks negatively correlated with the ΔHAZ score. Approximately 0.8% of the significantly affected peaks are within 2 kb of the TSS (*Inset*). Peaks in this set overlapped with 944 enhancers active in blood/immune cells. Motifs identified with these enhancers are shown by the logos, with E-values for their occurrence shown below each one. The motifs show statistically significant similarity to sites bound by ETS1 family and FOXO1 family transcription factors. (*G*) Top 10 reactome canonical pathways significantly associated with genes with H3K4me3 peaks positively correlated with ΔHAZ score identified with MSigDB. TAG, triacylglycerol. FDR-corrected q-value is shown.

To explore epigenetic changes associated with stunting at 1 y of age and their possible functional consequences, we identified differentially affected H3K4me3 peaks and computationally associated them with specific genes. Differential H3K4me3 peaks were first found by comparing the groups of control and stunted 1-y-old children. However, nearly all of the significantly different peaks were captured in the set of differential peaks associated with the child’s ΔHAZ score, and in addition, analysis of ΔHAZ-associated peaks uncovered 5,520 peaks not found in the simpler categorical comparison of control and stunted children (*SI Appendix*, Fig. S3*C*). H3K4me3 profiles of 1-y-old children were clearly distinguished by sex along the first principal component (PC) of a PC plot, and importantly, by ΔHAZ score across the second PC (Fig. 3*A*). Because the ΔHAZ score provides a quantitative measure of infant health in this setting, and because its use uncovered a richer set of differential peaks than the comparison of stunted and control samples, we focused on the differential H3K4me3 peaks associated with ΔHAZ score. (Few significantly affected peaks were identified in analyses of males and females separately, which we attribute to reduced statistical power associated with the smaller numbers of datasets for each sex individually.) H3K4me3 peaks that increased with increasing ΔHAZ score (peaks increased with improved health, which we refer to as “positive peaks”) tended to be larger than peaks that were increased in children with reduced ΔHAZ scores (peaks increased with poorer health, which we refer to as “negative peaks”) (Fig. 3*B* and *SI Appendix*, Fig. S3*D*). Positive peaks were in general located proximal to TSSs (Fig. 3 *C* and *D*), suggesting that their associated genes were more highly expressed in healthy compared with stunted children. In contrast, the statistically significant, albeit feeble, negative peaks were in general far removed from TSSs, consistent with the global redistribution of H3K4me3 signal described above (Fig. 3 *E* and *F*). Differential peak analysis was performed in a similar way for the H3K27ac data, which in contrast to H3K4me3 revealed very few significantly affected peaks (*SI Appendix*, Fig. S4). The H3K27ac results reinforce the conclusion that the reduced levels of H3K4me3 at canonical locations and redistribution to dispersed sites is a specific property of H3K4me3 in stunting and definitively not associated with reduced sample quality for stunted individuals.

Some H3K4me3 signals that accrued in stunted children away from TSSs may be functionally significant. Notably, the set of negative H3K4me3 peaks overlapped with 944 enhancers previously shown to be active in one or more blood or immune tissue/cell types (21) (*SI Appendix*, Table S3), and the overlapping enhancers were enriched in motifs recognized by ETS1 and FOXO1 family transcription factors (Fig. 3*F*), which play fundamental roles in immune cell development and function, as well as metabolism (22–25). Although the role of H3K4me3 at enhancers is less well understood (26), these results suggest that these enhancers may be more activated in stunted compared with control children. Paradoxically, of the 1,864 genes that were computationally or functionally linked to these 944 enhancers (21), only 13 genes have H3K4me3 peaks at the TSS that increased in stunted compared with control children, as would be expected if more highly activated enhancers led to increased transcription of enhancer target genes. This may suggest a global perturbation in transcriptional regulation in stunted children, some evidence for which is discussed below. The positive H3K4me3 peaks overlapped with 1,210 previously identified blood/immune cell enhancers, and consistent with their possible role in regulating gene expression in health, they were functionally associated with 3,507 genes with positive peaks at their TSSs (21) (*SI Appendix*, Table S3).

Because most positive peaks were proximal to TSSs, and the magnitudes of H3K4me3 peaks at TSSs tend to scale with the level of transcription (18), we focused on the genes associated with positive peaks for gene set enrichment analysis (GSEA). These genes comprised subsets with an array of enriched functional attributes consistent with the clinical presentation of stunting, including alterations in metabolism of proteins, lipids, and carbohydrates and multiple aspects of immune cell/system function (Fig. 3*G*). In addition, analysis of the genes that gave rise to enrichment in the reactome_generic_transcription_pathway category (Fig. 3*G*) revealed that many genes encoding components of the general transcription machinery for RNA polymerases I, II, and III had TSS-localized positive peaks. This included general transcription factors (e.g., TAFs, TFIIA, TFIIB, TFIIE, TFIIF, TFIIH, SL1) as well as many RNA polymerase subunits themselves. These results suggest that the overall transcriptional capacity of cells from stunted children may be reduced compared with normal controls. The elevated H3K4me3 levels at enhancers in stunted children (Fig. 3*F*) may indicate an inability to maintain the expression of core process genes in the face of reduced overall transcriptional capacity.

Consistent with GSEA, numerous fundamental pathways that drive cell growth and proliferation were computationally predicted to be differentially regulated based on the ΔHAZ score (*SI Appendix*, Fig. S5*A*). In addition, genes whose expression was predicted to be impacted were associated with a number of transcription factors which are known drivers of growth and immune processes (*SI Appendix*, Fig. S5*B*). Remarkably, the gene encoding an H3K4me3 demethylase (KDM5A) was identified as a factor with predicted altered activity in stunted compared with control children. As discussed below, RNA-seq data provide evidence that expression of an H3K4me3 demethylase is indeed altered in stunted individuals.

### Relationships Between Methylation Changes and Stature.

**Fig. 4.**
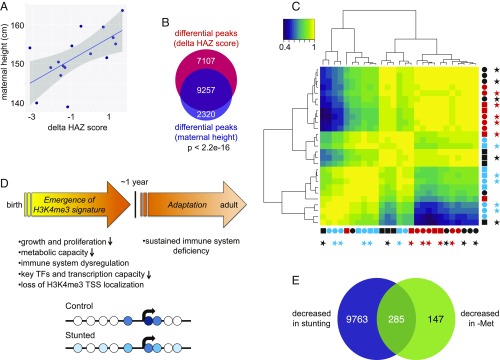
H3K4me3 pattern association with functionally relevant genes and changes over time. (*A*) Scatter plot showing ΔHAZ scores for children at 1 y of age versus the height of the child’s mother. (*B*) Comparison of H3K4me3 peaks in 1-y-olds correlated with ΔHAZ score versus the H3K4me3 peaks correlated with the height of the child’s mother. (*C*) Heat map showing the Pearson correlations between normalized H3K4me3 datasets from children at 18- and 52-wk and their mothers. The 18-wk datasets are labeled in red, 52-wk data in black, and maternal data in light blue. Circles indicate male and squares indicate female children; stunted children are denoted by stars. (*D*) Summary and working model of physiologic changes in cells from stunted children and H3K4me3 changes (*Upper*). Global H3K4me3 profile differences in control and stunted children at 1 y of age are depicted by the *Lower* schematic. Circles represent nucleosomes and the arrow represents the TSS of an average gene. Darker blue color signifies increased methylation level; white represents no H3K4 trimethylation. (*E*) Comparison of sets of genes with TSS-localized peaks decreased in human colon cancer cells minus methionine (green) (31) versus stunting at 1 y of age (blue; present study).

Adult height is determined by both genetic and environmental variables (27), and it can be affected by early life nutritional experience (28), which may extend to subsequent generations (29). In a previous study of this cohort, it was found that the height of a child’s mother is a predictor of whether the child will become stunted (19). Consistent with this, we observed that among the ChIP-seq samples analyzed, a child’s ΔHAZ score was correlated with the height of the child’s mother (Fig. 4*A*), and as a result, there was a substantial overlap in the differential H3K4me3 peaks associated with ΔHAZ score and those associated with maternal height (Fig. 4*B*). Independent analyses of the maternal datasets uncovered 603 H3K4me3 peaks associated with maternal height. Genes associated with these differential peaks were functionally enriched mainly in fundamental immune system categories, suggesting that the mother’s height is related at least in part to the overall functional health of her immune system. Maternal height appears most relevant in this predictive power, as there were no significant differential peaks identified in similar analyses performed using the mother’s weight or body mass index. In addition, 658 of 829 (79.4%) of the maternal genes associated with maternal height were also in the list of genes associated with maternal height in children at 1 y of age. This overlap is highly significant (*P* < 2.2e-16), indicating that H3K4me3 levels at a core set of genes provide a fingerprint of overall health in both mothers and children at 1 y of age. Defects in several differentially affected pathways in 1-y-olds including PI3K/AKT signaling, insulin receptor and IGF1 signaling, RANK signaling, growth hormone signaling, and p38 MAPK signaling (*SI Appendix*, Fig. S5*A*) have been implicated in determining stature, and in addition, 16 differentially affected H3K4me3 peaks overlap with single nucleotide polymorphisms linked to adult height through genome-wide association studies (30) (*SI Appendix*, Table S4). We suggest that these relationships reflect changes in gene expression across multiple tissues. If so, these results provide a plausible molecular explanation for why the affected individuals are short.

### Distinct Methylation Profiles in Children and Mothers.

To determine the global relationship between H3K4me3 modification patterns in children and their mothers, Pearson correlation coefficients were computed across all normalized peaks using datasets from nine children for whom we had results at both 18- and 52-wk time points as well as data from the child’s mother. The 18-wk datasets tended to cluster together, indicating that they resemble one another at a global level (Fig. 4*C*). The maternal datasets also tended to cluster together, indicating their overall similarity, but they mostly defined a separate cluster from the 18-wk cluster. This was in contrast to datasets from 1-y-old children, which were distributed between both the 18-wk and maternal clusters. Taken together, the results demonstrate that the pattern of H3K4me3 modification does not distinguish children at 18-wk, but rather a signature associated with stunting emerges by 1-y of age (Fig. 4*D*). The H3K4me3 stunting pattern can be described at a global level as resulting from the redistribution of signal from its typical locations proximal to TSSs to more distal sites, at least some of which are in enhancers and may have functional significance. There is no obvious similarity in the H3K4me3 patterns of 1-y-old children and their mothers, suggesting that at 1 y the epigenetic profile is in the process of maturing to the adult state, or that the profile at 1 y remains malleable to environmental or other stimuli beyond 1 y of age.

### Potential Causes of H3K4me3 Redistribution.

The computational prediction described above implicating a change in H3K4 demethylase activity in the global change in the H3K4me3 profile prompted us to explore available data to develop a model for how nutrients may alter histone demethylation. Notably, prior work has shown that histone demethylases respond transcriptionally to methionine starvation, and intriguingly, methionine starvation gives rise to a similar pattern of global H3K4me3 redistribution as we observe in stunted children (*SI Appendix*, Fig. S6) (31). Strikingly, TSS-proximal peaks affected in stunting and by methionine starvation are associated with an overlapping set of 285 genes with roles in amino acid transport, folic acid metabolism, DNA replication, and the cell cycle (Fig. 4*E*). Taken together, these results suggest that cells respond to limitation of one-carbon metabolites by regulating histone demethylase activity, and that an imbalance in demethylation enzymes can give rise to globally redistributed patterns of methylation like those we observe in stunted children.

### Gene-Expression Changes Associated with Stunting.

**Fig. 5.**
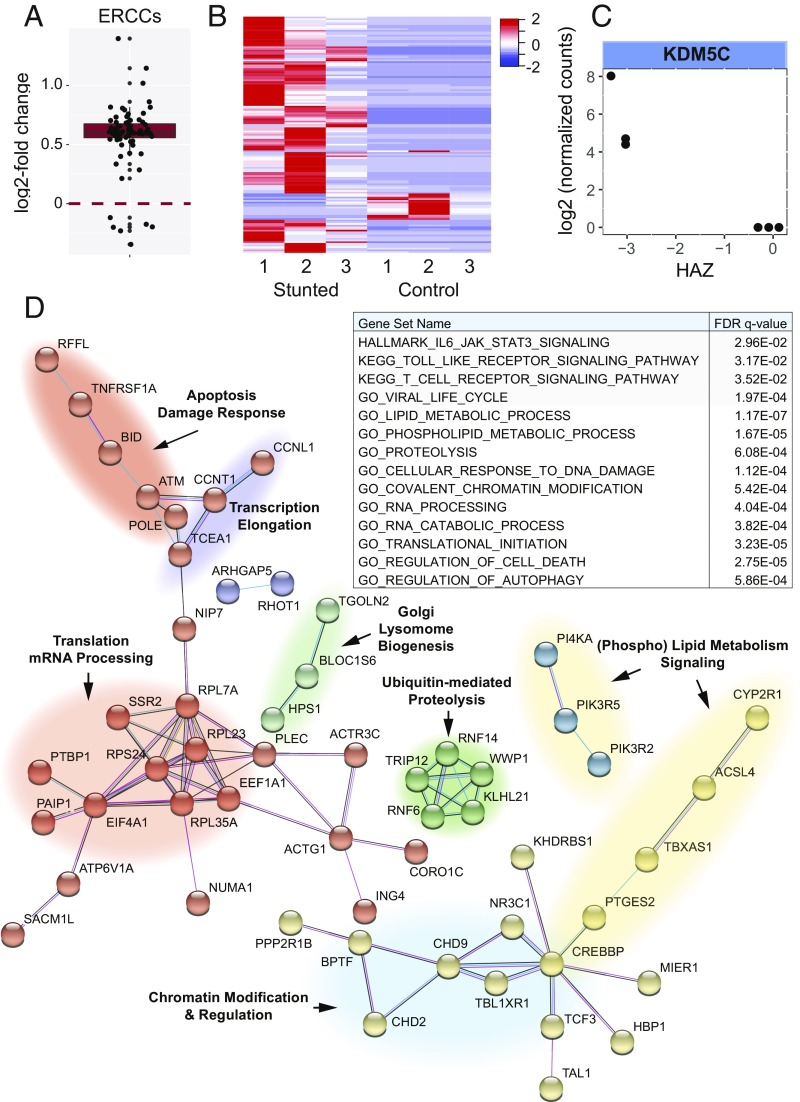
Gene-expression changes associated with stunting. (*A*) Box plot of log_2_ fold-changes in ERCC spike-in RNA levels per unit ΔHAZ score determined using DESeq2 default normalization. The deviation in log_2_ fold-change values from zero is highly significant (*P* < 2.2 e-16), which is consistent with increasing ribosomal RNA levels with health. (*B*) Heat map showing *z*-score–normalized levels of 143 differentially expressed RNAs (rows, gene names omitted for clarity) in samples from each of six female 1-y-old children. The samples are ordered by ΔHAZ score; three samples from children with HAZ scores < −2 were defined as phenotypically stunted, whereas three samples from children with HAZ scores > −2 were defined as controls. Ward.D clustering using a Pearson correlation similarity metric was applied to genes. (*C*) Log_2_ normalized transcript levels for the H3K4 demethylase gene KDM5C versus HAZ score. (*D*) Protein interaction network diagram obtained using STRING (58). The nodes represent protein products of differentially expressed transcripts versus ΔHAZ score. The network was determined using all STRING interaction sources except text mining and default parameters for interaction score. The protein–protein interaction *P* = 6.08e-4 for the network. Nodes are colored based on Markov clustering using an inflation parameter of 1.2. The colored clouds underlying different clusters identify functions and processes associated with the genes in that part of the network. Note that KDM5C was in a functional network of chromatin regulators but not directly linked to the network shown under these conditions. A partial list of MSigDB (53) gene set enrichment results is shown in the *Inset* table.

To begin to determine the relationship between the large-scale changes in H3K4me3 and gene expression, we performed RNA-seq using six PBMC samples from 1-y-old females with a range in ΔHAZ scores. As discussed above, one prediction from the H3K4me3 data was that there would be a global change in transcription associated with stunting. Using External RNA Controls Consortium (ERCC) spike-in RNAs added to total RNA samples from which ribosomal RNA was subsequently depleted before sequencing, we observed a significant shift in the normalized spike-in read counts that correlated with ΔHAZ score (Fig. 5*A*). This is consistent with increasing ribosomal RNA levels with health and fits with the well-established coordination in eukaryotic cells of ribosomal RNA synthesis, nutrient availability, and overall growth rate (32). The shift in spike-in reads with ΔHAZ score is also consistent in part with possible mRNA stabilization in response to stress, including nutrient limitation (33, 34). Using ERCC spike-in normalization, 143 differentially expressed RNAs were identified versus ΔHAZ score (Fig. 5*B* and *SI Appendix*, Table S5). Among them, we identified the H3K4 demethylase KDM5C, whose expression was increased with increasing degree of stunting (Fig. 5*C*). The observation of altered expression of KDM5C is notable for two reasons. First, it is consistent with the general model outlined above in which the global redistribution in H3K4me3 in stunted children may be driven by changes in methylation or demethylation enzymes. Second, a functional role for altered KDM5C expression is intriguing because KDM5C has been implicated in dampening expression by demethylating histone H3K4 at TSSs while having an activation function at enhancers (35). Thus, the biological role of KDM5C is consistent with it contributing to the observed shift in methylation away from TSSs and to distal sites, including regulatory regions. Importantly, loss of KDM5C did not impact overall levels of H3K4me3 (35), consistent with results here demonstrating redistribution of H3K4me3 without a significant change in overall methylation levels.

The differentially expressed RNAs were associated with numerous enriched gene categories consistent with predictions made using the H3K4me3 data, and moreover, ∼40% of the affected genes comprise a functional network whose submodules have functional attributes consistent with the body of work presented here (Fig. 5*D*). In addition to KDM5C, we found a collection of other differentially expressed chromatin regulators, as well as altered expression of genes implicated in RNA splicing/processing as well as translation. Genes associated with protein turnover and apoptosis/DNA damage were increased in expression in stunted individuals. Some genes in these categories are involved in autophagy/mitophagy and metabolic salvage pathways, consistent with nutrient limitation in stunting leading to activation of pathways aimed to recover or preserve nutrients by dismantling cellular structures or organelles. Lipid and phospholipid metabolic genes were also notably differentially expressed, consistent with the extraordinary leanness of stunted children activating pathways to mobilize any and all remaining reserves of fat. In addition to gene set enrichment identifying changes in fundamental aspects of cellular physiology, there was enrichment of various immune system categories as well (Fig. 5*D*, *Inset* table; discussed below). Importantly, the overlap in genes whose expression increased in health with those genes with significantly increased H3K4me3 in health was highly significant (*P* < 1.71e-9) (*SI Appendix*, Fig. S7), indicating that the levels of methylation were associated with commensurate changes in expression at a number of genes. Interestingly, the overlap in genes whose expression increased in stunting with H3K4me3 positive peaks was also highly significant (*P* < 3.08e-11); possible explanations for this are discussed below.

### Role for LDL Receptor 1 in a Mouse Model of Stunting.

**Fig. 6.**
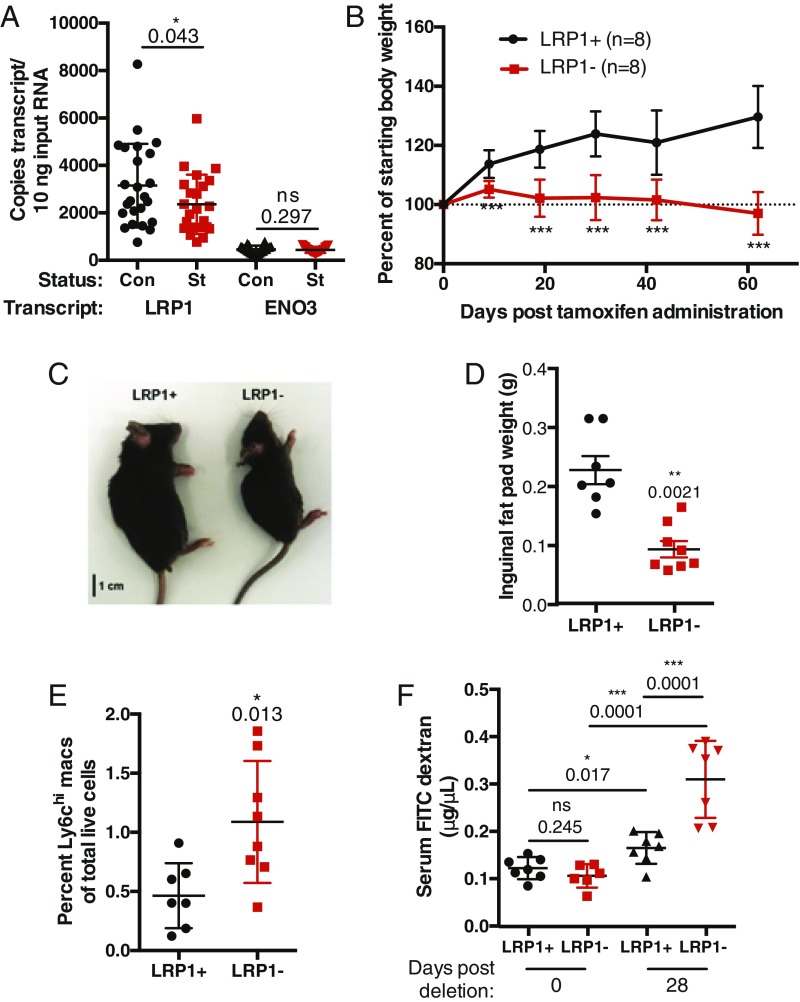
LRP1-deleted mice have a stunted phenotype and recapitulate pathophysiological aspects of stunting in humans. (*A*) LRP1 RNA levels in whole blood from 1-y-old control (Con) or stunted (St) children. ENO3 was chosen as an unaffected control. (*B*) Percentage weight gain in LRP-1^fl/fl^Cre-ERt2^+^ (*LRP1*−) and LRP-1^+/+^Cre-ERt2^+^ (*LRP1*^+^) mice after injection of tamoxifen. ***P < 0.001. (*C*) Appearance of *LRP1*^+^ and *LRP1*^−^ littermates 40 d postinjection of tamoxifen. (*D*) Inguinal fat pad weight in *LRP*^+^ and *LRP*^−^ mice. Each dot represents one animal. (*E*) Levels of Ly6C^hi^ inflammatory macrophages in intestinal lamina propria from *LRP1*^+^ and *LRP1*^−^ mice. (*F*) Serum FITC dextran levels in *LRP1*^+^ and *LRP1*^−^ mice at 0 and 28 d posttamoxifen treatment.

Next, we explored the genes with the most robustly affected H3K4me3 peaks at 1 y of age to identify candidates with possibly central roles in stunted children. The LDL receptor 1 (*LRP1*) gene was found among the top 0.5% of the ΔHAZ-associated peaks ranked by false-discovery rate (FDR)-corrected *P* values. LRP1 plays fundamental roles in endocytic trafficking, with a large number of known substrates, including apolipoprotein E, α2 macroglobulin, and numerous molecules involved in the immune response (36, 37). The key role of LRP1 in both lipid metabolism and immune responses, which were identified by RNA-seq, suggested that its altered expression could contribute to the stunted phenotype. We confirmed reduced expression of LRP1 in stunted children by droplet digital PCR using blood samples from a set of children in the same cohort (Fig. 6*A*). To determine the consequence of reduced LRP1 expression in the absence of other changes, we employed a tamoxifen-inducible LRP1 knockout mouse model. The induced deletion of LRP1 resulted in growth arrest (Fig. 6*B* and *SI Appendix*, Fig. S8 *A* and *B*) and *LRP1*^−^ mice were visibly smaller by day 40 posttamoxifen treatment (Fig. 6*C*) and had a markedly reduced inguinal fat pad (Fig. 6*D*). In addition, *LRP1*^−^ mice had increased levels of intestinal inflammatory macrophages (Fig. 6*E*) and compromised intestinal barrier integrity (Fig. 6*F*). The reduced body size, gut inflammation, and loss of intestinal barrier function observed in *LRP1*^−^ mice strongly parallel the clinical presentation of stunted children and suggest that reduced *LRP1* expression may be a driver of stunting in humans. *LRP1*^−^ mice had similar levels of ambulatory activity and despite their reduced size consumed more chow than *LRP1*^+^ controls (*SI Appendix*, Fig. S8*C*), indicating that their reduced size cannot be explained by differences in activity or food intake. There were also no significant overall changes in metabolic activity detected in comparing *LRP1*^+^ and *LRP1*^−^ mice (*SI Appendix*, Fig. S8 *D* and *E*).

## Discussion

### Global Changes in H3K4me3 Pattern in Stunted Children.

A major finding of this study is that H3K4me3 is redistributed from TSS proximal locations to ectopic sites in stunted children. This pattern would arise artificially if datasets from stunted children were simply noisier (of poorer quality) than the datasets obtained from control children, but we rule this out for six reasons. First, the datasets were obtained from coded samples, collected randomly, and selected for ChIP-seq in random order over about a 1-y period; thus, there is no basis for distinguishing the handling of the stunted versus control samples during the sample acquisition or data-generation stages. Second, because the total levels of H3K4me3 were not significantly different in stunted compared with control children (demonstrated independently by Western blotting and spike-in normalization), global decreases in H3K4me3 at canonical sites must be compensated by increases in signal elsewhere. Third, despite the globally reduced peak sizes at TSSs in stunted children, >90% of the peaks identified in control samples were independently identified in stunted samples, indicating that datasets from stunted children nonetheless contain robust localized H3K4me3 peak information. Fourth, we observed a quantitative relationship between thousands of H3K4me3 peaks and a child’s ΔHAZ score. Thus, H3K4me3 peak heights track incrementally along the continuum of measurements associated with how well a child grew during the first year; this cannot be plausibly explained by technical considerations. Fifth, differences in the composition or quality of the blood samples are not a factor as we found the same fraction of PBMC cell types in stunted and control samples (Fig. 2*I*). Finally, we find that the genome-wide pattern of H3K27ac is very similar in stunted and healthy children, demonstrating that the global change in H3K4me3 is not a general property of histone modifications, or even of another modification that tends to occur in the vicinity of H3K4me3. Taken together, we conclude that H3K4me3 signal is delocalized in stunted children from its canonical location at TSSs. As we described above, this altered pattern in stunting is also consistent with an independent study investigating the cellular response to methionine limitation. Interestingly, a similar pattern of methylation redistribution was observed in cells with a defect in methyltransferase targeting to chromatin (38). This global pattern of histone methylation change is also reminiscent of the global changes in chromatin structure induced in worms by early life mitochondrial stress and driven by methylation (39).

### Gene-Expression Changes in Stunting.

The functional categories associated with differential expression suggest strategies are employed in the cells of stunted individuals to retain or acquire essential limiting metabolites, particularly fat. The RNA-seq data also suggest regulation occurs at transcriptional, posttranscriptional, and translational levels, and is accompanied by or driven by changes in chromatin structure mediated by a network of chromatin-modifying enzymes. It is possible that the apoptotic factors whose expression increases in stunting reflect increased rates of apoptosis, but the collection of differentially expressed genes includes both activators and inhibitors of apoptosis; it is possible that nutrient scavenging pathways in stunted individuals trigger autophagy or mitophagy, leading to dismantling of organelles including mitochondria and that apoptosis occurs incidentally as a result. GSEA of both the H3K4me3 and RNA-seq data reveal highly significant changes in immune system genes, consistent with immune system dysfunction in stunted individuals. However, the complexity of differentially affected immune pathway activators and inhibitors makes specific molecular predictions of immune system dysfunction in stunting unreliable at this stage.

### Comparison of Differential H3K4me3 and Transcription Changes.

In overlap analyses of the differential H3K4me3 and RNA data, we identified a subset of genes with differential H3K4me3 peaks at the TSS with a change in methylation in the same direction as the change in expression (*SI Appendix*, Fig. S7). This overlap is highly significant statistically. Most of the other differentially expressed genes did not have a correlated change in a TSS localized peak, however. This makes the relationship between changes in methylation and changes in expression not straightforward to interpret, but it is consistent with numerous other studies demonstrating that while H3K4me3 levels are relatively good predictors of expression, defects in the methylation/demethylation machinery can give rise to gross changes in methylation that are associated with more circumscribed changes in expression (40). It is also possible that changes in gene expression are secondary to changes in methylation elsewhere, or vice versa. Along these lines, the overlap between genes whose expression increased with stunting but whose H3K4me3 peak increased with health was also highly significant (*SI Appendix*, Fig. S7). Taken together, the RNA-seq data provide strong support for the main biological conclusions derived from the H3K4me3 data, including reduced activity of fundamental growth pathways, a role for an H3K4me3 demethylase, defects in core metabolic and immune system pathways, and a likely global change in ribosomal RNA levels. Obtaining samples from this vulnerable population is extremely challenging; when it is possible to analyze additional samples using RNA-seq it is likely that many more gene-expression changes will be discovered.

### Contribution of *LRP1* to Stunting Phenotype.

Although LRP1 was known to be involved in metabolic and immune cell function, a role for systemic LRP1 in stunting was not anticipated. Phenotypic effects of LRP1 loss in mice were reported to vary greatly—from weight loss to, surprisingly, weight gain—depending on the particular tissue in which LRP1 depletion was induced or measured (41–45). Moreover, the suggestion that LRP1 levels in the whole animal drive the stunted phenotype is not based solely on the reduced size of *LRP1*^−^ mice; importantly, loss of LRP1 led to increased intestinal inflammation and permeability, two hallmarks of the stunted state. These results suggest that reduced LRP1 expression contributes to stunting in humans. The absence of gross metabolic changes in *LRP1*^−^ mice suggests the possibility that stunting is primarily triggered by defects in immune responses rather than nutritional limitation per se.

## Methods

Details on experimental procedures are available as *SI Appendix*, *Experimental Methods and Materials*.

### Human Subjects.

The study was approved by the Ethical Review Board of ICDDR,B (FWA 00001468) and the Institutional Review Boards of the University of Virginia (FWA 00006183) and the University of Vermont (FWA 00000727). Within 7 d after giving birth, screening for eligibility and study consenting occurred in the household by trained Field Research Assistants. Informed consent was obtained for all participating mothers and infants (trial registration: ClinicalTrials.gov NCT01375647).

### ChIP-Seq.

Peripheral blood cell samples were obtained from individuals at 18 and 52 wk of age and from mothers enrolled in the PROVIDE Study (17). Following formaldehyde fixation, chromatin isolation, and shearing, H3K4me3- or H3K27ac-associated DNA fragments were isolated by immunoprecipitation using anti-H3K4me3 antibodies (Cell Signaling Technologies) or H3K27ac (Diagenode) antibodies and libraries were constructed using the Illumina TruSeq ChIP Library Preparation Kit. Four datasets were also obtained post hoc using chromatin samples to which sonicated *Drosophila* chromatin (Active Motif #53083) was added for spike-in normalization (46). Sequencing of H3K4me3 libraries was performed on an Illumina MiSeq instrument. Multiplexed H3K27ac libraries were sequenced using an Illumina NextSeq500 instrument; both sequencers are in the University of Virginia DNA Sciences Core Facility.

### Analysis of ChIP-Seq Datasets.

Raw H3K4me3 sequence reads were mapped to the hg19 version of the human genome using Bowtie 1.0.0 (47); the resulting files were processed to remove unmapped reads and then converted to bam format using SAMtools v0.1.19-44428cd (48). Peaks of H3K4me3 enrichment were called using MACS-1.4.2 (49) with a sex-matched input dataset as control and with default parameters (50). Spike-in datasets were mapped in addition to the *Drosophila* dm6 genome to obtain read counts for spike-in normalization. Count tables consisting of read counts for each dataset in the union set of all called peaks were used as input to DESeq2 (51). Differentially affected peaks were identified using DESeq2 and with default normalization or user-specified normalization based on spike-in read counts or an exponential fit of the read count distributions in 150-bp bins tiled across the human genome. Significantly affected peaks were assigned to genes using GREAT (52), and gene set and pathway enrichment was performed using MSigDB and GSEA (53) and IPA (QIAGEN Redwood City, https://www.qiagenbioinformatics.com/). H3K27ac datasets were analyzed in the same way except that reads were mapped using bowtie2-2.2.6 (54) and peak calling was performed using MACS2-2.1.1.20160309 (49).

### RNA-Seq.

Total RNA was obtained from six PBMC samples from 1-y-old females with a range in ΔHAZ scores. ERCC spike-in RNA (Illumina) was added to aliquots of total RNA per instructions of the manufacturer, ribosomal RNA was then depleted using the RiboZero gold kit and libraries were constructed using the NEBNext Ultradirectional RNA Lib Prep Kit (Cat #E74205) and NEBNext Multiplex Oligos (Cat#E73355). The 75-bp paired-end reads were obtained from the NextSeq500 instrument described above. Raw reads were assessed by FASTQC (https://www.bioinformatics.babraham.ac.uk/projects/fastqc/) and mapped to the hg19 reference genome using HISAT2 (55). RNAs were then assembled and quantified using StringTie (56) and differential analysis was performed on the resulting transcripts count table using DESeq2 (51).

### Molecular Biological Analysis of Human Samples.

Western blots were performed using the same chromatin extracts used for ChIP-seq. Cross-links were reversed by heating, and chromatin proteins were resolved on 4–20% gradient polyacrylamide gels and transferred to Immobilon-P^SQ^ (Millipore #ISEQ00010), as described previously (57). Blots were probed with rabbit anti-H3K4me3 C42D8 (Cell Signaling #9751) or rabbit anti-H3 C-terminal antibody (Active Motif #39163). Digital droplet PCR was performed on total RNA extracted from whole blood using PrimePCR ddPCR expression probe assays implemented on a QX200 Droplet Digital PCR System (Bio-Rad) and the results were analyzed using QuantaSoft software (Bio-Rad).

### *LRP1*^−/−^ Mice.

*LRP1*^*fl*/fl^ Cre^+^ were administered tamoxifen (Sigma-Aldrich) intraperitoneally three times at a dose of 75 mg/kg body weight every 10 d. Depletion of LRP1 protein in *LRP1*^−/−^ mice was confirmed by LRP1 immunoblotting using extracts from brain, liver, and lung tissue. Body weight was measured using a digital scale. Mice were individually housed for measuring food intake, and chow consumed was measured by averaging the difference between the weight of chow before and after a 24-h period over 3 d. Fat and lean mass were measured using an EchoMRI-500 instrument as recommended by the manufacturer. Metabolic caging experiments were conducted using an Oxymax-Comprehensive Animal Monitoring System (Columbus Instruments) as recommended by the manufacturer. All mouse procedures were approved by the Institutional Animal Care and Use Committee of the University of Virginia.

H3K4me3 ChIP-seq data and metadata are available from dbGaP under accession no. phs001073.v1.p1. RNA-seq data and metadata are also available from dbGaP under accession no. phs001665.v1.p1.

## Supplementary Material

Supplementary File
