# Supplementary material for: Histone H3 lysine 4 methylation signature associated with human undernutrition
Source: Proc Natl Acad Sci U S A. 2018 Nov 12;115(48):E11264–73. doi: 10.1073/pnas.1722125115 (PMC6275549; doi:10.1073/pnas.1722125115)
Supplement: Supplementary File [file pnas.1722125115.sapp.pdf]

PNAS
www.pnas.org

Supplementary Information for

"Histone H3 lysine 4 methylation signature associated with human undernutrition"

Robin Uchiyama, Kristyna Kupkova, Savera J. Shetty, Alicia S. Linford, Marilyn G. Pray-Grant, Lisa E. Wagar, Mark M. Davis, Rashidul Haque, Alban Gaultier, Marty W. Mayo, Patrick A. Grant, William A. Petri, Jr., Stefan Bekiranov, and David T. Auble

Email: auble@virginia.edu

This PDF file includes:

Supplementary text
Figs. S1 to S8
Tables S1 to S5
References for SI

www.pnas.org/cgi/doi/10.1073/pnas.1722125115

Supplementary Information Text

Section 1: Experimental Methods and Materials

Isolation of peripheral blood mononuclear cells (PBMCs) from human whole blood

PBMCs were isolated at icddr,b (International Centre for Diarrhoeal Disease Research, Bangladesh) in Dhaka, Bangladesh. Peripheral blood samples from 18-week-old and 52-week-old children and from their corresponding mothers enrolled in the PROVIDE study (1) were collected in tubes supplemented with anticoagulants (e.g. heparin, EDTA, citrate, ACD-A or citrate phosphate dextrose). Blood samples were processed within 8 hours of collection. To isolate PBMCs, per 2 mL blood: blood was diluted with 2-4X the blood volume of buffer (PBS supplemented with 2% heat-inactivated FBS ((cat #D-S11550, Atlanta Biologicals, Flowery Branch, GA, USA); FBS was heat-inactivated by thawing to room temperature followed by incubation at 56ºC for 30 min and then cooling). Approximately 6 mL of diluted cell suspension was carefully layered over 3 mL Ficoll-Paque (GE Healthcare Life Sciences, Pittsburgh, PA, USA) in a 15 mL conical tube and centrifuged at 1000 x g for 15 min at 22ºC in a swinging-bucket rotor without brake. The upper plasma layer was aspirated, and the mononuclear cell layer was carefully transferred to a new 15 mL conical tube. The sample volume was adjusted to approximately 10 ml with PBS, mixed, and centrifuged at 400 x g for 10 min at 22ºC. The cells were counted using a hemocytometer, and the washing step was repeated in order to remove any residual Ficoll. The supernatant was carefully removed and PBMCs were resuspended with gentle swirling in 1 mL freezing medium (90% heat-inactivated FBS with 10% DMSO (cat #D5879, Sigma-Aldrich, St. Louis, MO, USA)). Cells were placed on ice and transferred into a cryopreservation vial by slowly pipetting. The cryovial was capped, placed into a pre-cooled freezing container filled with isopropanol (such as a "Mr. Frosty" (cat #5100-0001), Thermo Fisher Scientific, Waltham, MA, USA), placed at -80ºC for 24 h, then moved to storage in liquid N_2. Frozen vials containing sample PBMCs were shipped to the University of Virginia using a dry shipper charged with liquid N_2, and vials were subsequently stored at -80ºC prior to crosslinking.

PBMC Crosslinking and Lysis

Frozen PBMC samples stored at -80°C were crosslinked and lysed using protocols adapted from the NIH Roadmap Epigenomics Mapping Consortium [http://www.roadmapepigenomics.org/protocols] and Gilfillan et al (2012) (2). Work with unfixed cells was performed in a biological safety cabinet. Each cryovial of frozen PBMCs contained $4x10^6$ to $2x10^7$ cells. Typically four specimens were processed at a time. Cells were thawed by warming the vials in gloved hands, contents (~1 mL) were immediately transferred to 15 mL conical tubes and tubes placed on ice. Vials were rinsed once with 1 mL ice cold PBS, and the rinse added to each corresponding 15 mL tube. Conical tubes were centrifuged in a tabletop centrifuge at 1500 x g for 5 min at 4°C. Supernatants were aspirated, and cell pellets rinsed with 1 mL ice cold PBS. Conical tubes were centrifuged again at 1500 x g for 5 min at 4°C and the supernatant aspirated. To crosslink samples, 5 mL room temperature FIX solution (1% formaldehyde in DMEM (cat #11995-065, Gibco/Thermo Fisher Scientific, Waltham, MA, USA)) was added to each tube, and tubes were incubated for 10 min at room temperature with gentle inversion. Fixation was stopped by adding 5 mL room temp STOP-FIX solution (250 mM glycine in PBS) to each tube and incubating tubes with gentle inversion for 5 min at room temp. PBMCs were pelleted by centrifugation at 4000 rpm for 5 min at 4°C and supernatant was aspirated. Cell pellets were rinsed with 5 mL ice cold PBS, tubes were centrifuged at 4000 rpm for 10 min at 4°C, and supernatant aspirated, with any remaining PBS

removed as carefully as possible. One mL of 4°C SDS Lysis Buffer (1% SDS, 10 mM EDTA pH 8.0, 50 mM Tris HCl pH 8.1, 1 cOmplete protease inhibitor tablet, EDTA-free (cat #11873580001, Roche, Indianapolis, IN, USA) per 20 mL buffer) was then added to each cell pellet, and pellets were suspended by pipetting. If SDS precipitates were present in the buffer, the buffer tube was warmed until the precipitates were solubilized. The resulting cell lysates were transferred to 1.5 mL microfuge tubes and incubated on ice a minimum of 10 min before sonicating.

PBMC Sample Sonication

Lysed PBMCs were sonicated using conditions adapted from Adli & Bernstein 2011 (3) using a Branson Digital Sonifier Model 250 (Branson Ultrasonics Corporation, Danbury, CT, USA) to generate fragment sizes in the range of 150-200 bp to 500-600 bp as visualized by 2% agarose gel electrophoresis. Samples were kept on ice at all times. Each 1.5 mL microfuge tube was placed in a wet ice bath in a plastic beaker during sonication, with the probe tip submerged as deeply as possible in the sample tube without touching tube bottom or sides. Settings were: 10 min of total sonication, 0.7 sec "on", 1.3 sec "off", 40% power. Sonication was paused after every minute of sonication to add ice and/or remove water from the wet ice bath. The probe was rinsed thoroughly between samples with 70% ethanol followed by sterile distilled water. After all samples had been sonicated, sample tubes were microfuged at 4°C for 10 min at maximum speed to pellet insoluble debris. If SDS precipitates were visible in sample tubes, tubes were warmed in gloved hands to resolubilize SDS before centrifuging. Supernatants were removed to new 1.5 mL microfuge tubes, placed on ice, and protein concentration of the resulting chromatin solution was measured with the Bradford Protein Assay (cat #5000006, Bio-Rad, Hercules, CA, USA) using BSA as the standard. Lysates were frozen on dry ice and stored at -80°C.

Chromatin Immunoprecipitation (ChIP)

Samples were kept at 4°C at all times. Sonicated chromatin lysate aliquots of 100 μg total protein were used for each ChIP sample. For libraries containing spike-in chromatin, 0.2 μg of sonicated Drosophila chromatin (Active Motif #53083) was also added. Input controls consisted of 40 μL aliquots of sonicated chromatin lysate, which were used to check DNA fragment size, and after processing, in qPCR to estimate ChIP sample enrichment. ChIP and Input samples were diluted 1:5 with ice cold ChIP Dilution Buffer (0.01% SDS, 1.1% Triton X-100, 1.2 mM EDTA (pH 8.0), 16.7 mM Tris-HCl pH 8.1, 167 mM NaCl, 1 cOmplete protease inhibitor tablet, EDTA-free (cat #11873580001, Roche, Indianapolis, IN, USA) per 20 mL buffer). Diluted input samples were stored at 4°C prior to crosslinking reversal. Anti-H3K4me3 Ab (12.5 μl per 100 ug chromatin protein solution, cat #9751, Cell Signaling Technology, Danvers, MA, USA) was added to each sample and incubated overnight at 4°C with rotation. After overnight incubation, 50 μl of a 1:1 mixture of Dynabeads Protein A and G (cat #10001D and #10003D, Life Technologies/Thermo Fisher Scientific, Waltham, MA, USA) was used to capture the DNA-protein-antibody complexes. Beads were washed twice with 2X the original bead volume of cold ChIP Dilution Buffer. The beads were resuspended in 1X bead volume of cold ChIP Dilution Buffer. Washed beads were added to each sample tube that had incubated overnight with antibody at 4°C. Sample tubes were incubated with beads for 2 hours at 4°C with rotation and then washed a total of 6 times: (a) 2 washes with ice cold Low Salt Immune Complex Wash Buffer (0.1% SDS, 1% Triton X-100, 2 mM EDTA, 20 mM Tris-HCl pH 8.1, 150 mM NaCl), (b) 2 washes with 1 mL ice cold LiCl Immune Complex Wash Buffer (0.25 M LiCl, 1% IGEPAL CA-630/Nonidet P-40, (cat #I1112 Spectrum Chemical Mfg. Corp., New Brunswick, NJ, USA, or cat #I8896, Sigma-Aldrich, St. Louis, MO, USA), 1% sodium deoxycholate (Sigma-Aldrich, St. Louis,

MO, USA, cat #D6750), 1 mM EDTA, 10 mM Tris-HCl pH 8.1), and (c) 2 washes with 1 mL ice cold TE buffer pH 8. After washing, bead pellets were suspended in 125 µL room temperature Elution Buffer (10 mM Tris-HCl pH 8.1, 1 mM EDTA pH 8.0, 1% SDS, 150 mM NaCl) with 5 mM DTT. Samples were incubated at 65°C for 10 min, the supernatant recovered, and the elution repeated. Input samples were diluted with Elution Buffer as well, and crosslinks in all samples were reversed by incubation at 65°C overnight.

After the 65°C overnight incubation, 250 µL of Proteinase K in Elution Buffer without DTT (5 µL of 20 mg/mL Proteinase K (cat #25530015, Invitrogen/Thermo Fisher Scientific, Waltham, MA, USA) and 245 µL of Elution Buffer without DTT) were added to each sample and incubated at 37°C for 2 hr. Samples were then extracted twice with Tris-saturated phenol pH 7.9 (cat #BP1750I100, Fisher BioReagents/Thermo Fisher Scientific, Waltham, MA, USA) and once with chloroform:isoamyl alcohol (24:1). The aqueous samples were ethanol precipitated, washed with 70% ethanol and resuspended in 50 µL of 1X TE pH 8.0 containing 0.2 mg/ml RNase A (cat #EN0531, Thermo Fisher Scientific, Waltham, MA, USA), then incubated at 37°C for 30 min prior to storing at -80° C. Recovered DNA samples were quantified using a Qubit fluorometer (Life Technologies/Thermo Fisher Scientific, Waltham, MA, USA) and the Qubit dsDNA High Sensitivity Assay kit (cat #Q32854 (reagents), #Q32856 (assay tubes), Life Technologies/Thermo Fisher Scientific, Waltham, MA, USA) as recommended by the manufacturer.

ChIP-seq Library Construction and Sequencing

H3K4me3 ChIP-seq libraries were constructed using 5-10 ng ChIP or input DNA with the Illumina TruSeq ChIP Library Preparation Kit (cat #IP-202-1012, Illumina, San Diego, CA, USA) as recommended by the manufacturer with the following exceptions. Gel slices included 200-650 bp DNA fragments, and two MinElute columns were used rather than one. ChIP-Seq and input library DNA was quantitated using the Qubit fluorometer as described above, and the size distributions of fragments in completed libraries were assessed using DNA 1000 microfluidics chips (cat #5067-1504, Agilent Technologies, Santa Clara, CA, USA) on an Agilent 2100 Bioanalyzer (Agilent Technologies, Santa Clara, CA, USA). Enrichments for ChIP-Seq libraries were estimated by qPCR using primer sets for enriched and un-enriched control genomic regions, and each ChIP-seq library had fold enrichments consistent with published guidelines (4, 5). Libraries were sequenced on an Illumina MiSeq instrument (Illumina, San Diego, CA, USA) in the University of Virginia DNA Sciences Core Facility. H3K27ac libraries were constructed as above except using an H3K27ac antibody from Diagenode (Cat# C15410-196, lot# A1723-0041D) and in addition, 0.02% (microgram/microgram) Drosophila spike-in chromatin was added prior to immunoprecipitation. Multiplexed H3K27ac samples were sequenced in the UVA Biomolecular Core Facility using an Illumina NextSeq500 system with high capacity cartridge.

RNA-seq library construction and sequencing

We obtained six PBMC samples from females at one year of age residing in Dhaka. Total RNA was extracted using the Zymo research Quick-RNA miniprep kit (Cat# 11-327). ERCC spike-in RNAs (Cat#4456740 Illumina) were added to 1mg of total RNA as recommended by the manufacturer, and ribosomal RNA depletion was performed using the RiboZero gold kit (Cat#MRZG126). cDNA libraries were prepared with 20ng of rRNA-depleted RNA using the NEBNext Ultradirectional RNA Lib Prep Kit (Cat #E74205) and NEBNext Multiplex Oligos (Cat#E73355). Library quality was assessed on the Agilent 2100 Bioanalyzer and 75bp paired-end reads were obtained in a single

multiplexed run on an Illumina NextSeq500 system with high capacity cartridge in the UVA Biomolecular Core Facility.

Droplet digital PCR

Five hundred µL of blood collected from stunted (HAZ ≤ -2) and non-stunted (HAZ > -2) children at 52 weeks of age enrolled in the PROVIDE cohort (1) was suspended in 4 mL of TRIzol LS, mixed gently, and frozen for later use. Water (833 µL) was added to maintain the 3:1 volume of TRIzol LS to sample, and 2 mL of sample was purified for LRP1 expression analysis. RNA was isolated from whole blood suspended in TRIzol LS (Thermo Fisher Scientific), according to the manufacturer's instructions for RNA isolation from biological fluids with small sample quantities. The sample was then treated with DNase I (Qiagen), and RNA was purified and concentrated using RNeasy MinElute Cleanup Kit (Qiagen). LRP1 and ENO3 transcripts were amplified using PrimePCR ddPCR expression probe assays (assay ID dHsaCPE5026596 for LRP1 and dHsaCPE5045082 for ENO3, Bio-Rad) and the one-step advanced RT-ddPCR kit for probes (Bio-Rad). Droplets were generated, and samples were run on the QX200 Droplet Digital PCR System (Bio-Rad). Samples were analyzed using QuantaSoft software (Bio-Rad). LRP1 and ENO3 expression analysis was performed in triplicate for each sample, and each data point represents the average transcript number.

Western blotting

Formaldehyde crosslinked cells were resuspended in 1% SDS, 10mM EDTA, 50mM Tris-HCl (pH 8.1). Crosslinks were then reversed by incubating cell suspensions in 1x Laemmli buffer at 100°C for 30 mins. Extracts were then clarified by centrifugation at 21,000 x g for 10 mins. Twenty (20) micrograms of total protein, estimated by Pierce BCA Protein Assay (ThermoFisher Scientific #23225), were separated on 4-20% gradient gels and transferred to Immobilon-P^{SQ} membrane (Millipore #ISEQ00010) according to Vettese-Dadey et al (1996) (6). Membranes were blocked in 3% BSA (w/v) in TBS-T and incubated with rabbit anti-H3K4me3 C42D8 (Cell Signaling #9751) at a dilution of 1:1,000 in TBS-T. Secondary antibody was either ECL Plex goat anti-rabbit IgG-Cy5 (GE Healthcare #PA45011) diluted 1:2,500 in TBS-T or ECL donkey anti-rabbit IgG-HRP (GE Healthcare #NA934) diluted 1:10,000 in TBS-T. Total H3 from the same extracts was measured using a rabbit anti-H3 C-terminal antibody (Active Motif #39163) diluted 1:10,000 in TBS-T as the primary antibody. Levels of H3Kme3 were quantified relative to total histone H3 by fluorescent western blotting detected using a Typhoon Trio laser scanner. Data are presented for every 52-week sample for which there was sufficient material remaining after ChIP-seq analysis to include on the blots.

Flow cytometry

Cryopreserved PBMC samples from a subset of 53-week-old Bangladeshi children were thawed into complete RPMI medium containing 10% FBS. Cells were rested for one hour in the presence of benzonase (Sigma) at 37°C for one hour then washed twice in FACS buffer (PBS with 0.1% bovine serum albumin, 2 mM EDTA, and 0.05% sodium azide). Cells were stained with the following anti-human antibodies in the presence of Fc receptor block and live/dead aqua zombie (all Biolegend) for 30 minutes: FITC-CD20, PerCP-Cy5.5-CD33, PE-Cy7-CD27, APC-CD38, Alexa700-CD14, APC-Cy7-CD3, Pacific Blue-CD16, Brilliant Violet 650-CD19. Samples were tested for either surface or intracellular LRP1 protein expression. Surface staining was done using anti-LRP1-PE antibody (eBioscience). For intracellular LRP1 staining, cells were fixed, permeabilized, and stained with anti-LRP1 (Abcam) followed by an anti-rabbit secondary PE antibody. After washing, data was collected using an LSRII instrument (BD) and data analysis was performed using Flowjo (Treestar).

Samples that showed few intact cells (<= 30% of total events) or few live cells (<= 60% of singlets) were excluded from the analysis. Subjects were defined as stunted if HAZ score at 53 weeks was below or equal to -2.

Mice and whole-body LRP1 deletion

All mouse procedures were approved by the Institutional Animal Care and Use Committee of the University of Virginia. LRP1 floxed, UBC-Cre-ERT2+ (LRP1$^{fl/fl}$ Cre+) and LRP1 floxed, UBC-Cre-ERT2- (LRP1$^{fl/fl}$ Cre-) mice on a C57BL/6 background were bred and maintained at the University of Virginia. The mice used to generate breeder and experimental mice, UBC-Cre-ERT2 and LRP1 floxed mice, were originally obtained from Jackson Laboratories and Joachim Herz of the University of Texas Southwestern Medical Center, respectively. To whole-body delete LRP1, LRP1$^{fl/fl}$ Cre+ were administered tamoxifen (Sigma-Aldrich) 3 times at a dose of 75 mg/kg body weight via the intraperitoneal route every 10 days. Depletion of LRP1 protein in LRP1-/- mice was confirmed by LRP1 immunoblotting using extracts from brain, liver, and lung tissue (not shown). Body weight was measured using a digital scale. Mice were individually housed for measuring food intake, and chow consumed was measured by averaging the difference between the weight of chow before and after a 24-hour period over 3 days.

Intestinal lamina propria isolation, cell staining and flow cytometry

Small intestinal lamina propria cells were isolated as previously described (7). The cell viability of 1 million cells/sample was assessed using Zombie Aqua dye (Biolegend). The cells were Fc receptor blocked using an anti-mouse CD16/32 antibody (Biolegend) and stained using the following anti-mouse antibodies to distinguish between innate and adaptive immune cells: CD11c-BV421 (Biolegend), CD4-BV605 (Biolegend), Ly6C-FITC (BD), CD3e-PerCP-Cy5.5 (Biolegend), SIGLEC F-PE (BD), Ly6G-PE-Cy7 (Biolegend), CD11b-APC (eBioscience), CD8a-AF700 (eBioscience), and CD45-APC-Cy7 (Biolegend). The samples were fixed using fixation buffer (Biolegend), assayed on a flow cytometer (LSRFortessa, BD), and analyzed using FlowJo software (Tree Star). To determine the percent of Ly6C^{hi} (inflammatory) macrophages of total live cells, single cells were gated on viability, CD45+, CD11b+ CD11c-, Ly6G-, and Ly6C^{hi}. In the CD11c-, CD11b+ gate, cells were further distinguished by expression of Ly6C and Ly6G. Of the Ly6G negative population, three populations were identified, Ly6G- Ly6C-, Ly6G- Ly6C^{low} and Ly6G-, Ly6C^{hi} by mean fluorescence intensity. Classification of Ly6G- Ly6C^{hi} cells as inflammatory monocytes was based on prior work (8, 9).

Intestinal permeability assay

Prior to the administration of FITC dextran (Sigma Aldrich), mice were fasted for at least 4 hours and then fed FITC dextran in PBS (Thermo Fisher Scientific) at a dose of 40 mg/100 g body weight by oral gavage. Four hours after FITC dextran administration, mice were bled, serum was obtained (BD Microtainer, BD), and serum was assayed for fluorescence using Synergy H4 microplate reader (BioTek).

Fat and lean mass determination

Fat and lean mass were assayed before and after LRP1 deletion using the EchoMRI-500 (EchoMRI) according to the manufacturer’s instructions.

Metabolic caging

Volume of O_2 consumed, volume CO_2 expelled, respiratory exchange ratio, and activity in the X and Y direction were measured and calculated using the Oxymax-Comprehensive Animal Monitoring System (Columbus Instruments), according to the manufacturer's instructions.

Section 2: Computational Methods

Power and sample size estimates for H3K4me3 ChIP-seq analysis

In order to estimate the sample size, we re-analyzed a DNA methylation data set generated by Khulan et al (10) in which Gambian women were given a supplement, UNIMMAP tablets, before pregnancy, followed by FeFol once pregnant and compared to control mothers who were given a placebo. Circulating blood was collected from supplement-treated and control mothers' infants at 9 months of age and their DNA was analyzed using Illumina HumanMethylation27 BeadChip microarrays. There were 5 female and 4 male infants from treated mothers and 8 female and 7 male infants from control mothers. We quantile normalized the fraction of methylated alleles, beta; calculated the mean and standard deviation of the difference between treated and control betas; calculated the pooled variance; and fit the pooled variance to an inverse gamma distribution (i.e., determined the shape and scale parameters of the inverse gamma distribution). We input these results into the R package *ssize.fdr* (11) (12), which calculates the sample size for fixed power, false discovery rate (FDR) and proportion of non-differentially methylated loci. We estimated that we had 90% power to detect 138 differentially methylated loci (i.e., proportion of non-differentially methylated loci equal to 0.995) at a 5% FDR using a two-sided t-test with 12 infant female control and treatment samples or 16 infant male control and treatment samples.

Processing of ChIP-seq datasets

H3K4me3 ChIP-seq datasets obtained from 21 children at 18 weeks of age and 16 children at 52 weeks of age, and included samples from boys and girls in roughly equal numbers and from control children and children who were or became stunted (height-for-age (HAZ) z-score score < -2) at one year of age (Table S1). In addition, datasets were obtained from 24 mothers of children in the same cohort. Samples were chosen based on these phenotypic attributes from among those not otherwise obligated for other investigations as part of the PROVIDE Study (1). We also obtained four input datasets from children at each age (samples from 2 males and 2 females, one stunted and one control child for each sex) plus two input datasets for the maternal samples, one from a mother of a control boy and one from a mother of a control girl. After these datasets were generated, to validate the normalization strategy, we also generated and analyzed H3K4me3 datasets from four 52-week samples with Drosophila spike-in chromatin as described below. We obtained ~30M single-end, 151 bp, raw reads from each library.

Raw reads in FASTQ format were mapped to the hg19 human genome using Bowtie-1.0.0 (13). Excluding hardware-specific arguments for improved speed and efficiency, default settings were used with the following exceptions: -m 3 --best --strata. The resulting SAM files were converted to BAM, unmapped reads were removed and then the files were sorted and indexed using SAMtools v. 0.1.19-44428cd (14). Spike-in datasets were mapped to the hg19 and Drosophila dm6 genomes using Bowtie2-2.2.6 (15) with default mapping parameters, then converted to BAM, unmapped reads were removed and the mapped reads were sorted and indexed using SAMtools as for the other datasets. Sorted and indexed BAM files were converted to BigWig using BEDtools-2.18.2 bamtobed and genomecov (16) and the bedGraphToBigWig converter (17). The resulting BigWig files were used for browsing the data with IGV (18). The browser screenshot in the main text was obtained by scaling sorted and indexed BAM files based on the total mapped read counts from each of the individual

datasets using BEDTools genomecov with an appropriate value for the -scale argument. Peaks of H3K4me3 enrichment were identified in each dataset using MACS-1.4.2 (19) with a sex-matched input dataset as control and with default parameters as detailed previously (20). Approximately 25,000 H3K4me3 peaks were identified for each dataset.

Datasets from children at 18 and 52 weeks, as well as maternal data, were first analyzed independently. Non-redundant lists of all peaks identified in one or more datasets were generated and read counts for each dataset distributed to each of the non-redundant genomic intervals using BEDTools-2.18.2 merge and multicov (16) with default parameters. The resulting tables of read counts were used as input to DESeq2 (21) for identification of differentially affected peaks. DESeq2 was implemented for differential analysis based on phenotypic differences (e.g. control versus stunted datasets) as well as biomarkers including HAZ score, delta HAZ, maternal height, and other biomarkers collected for individuals enrolled in the PROVIDE Study (1). Additional details regarding implementation of DESeq2 and differential peak analysis are described below.

H3K27ac ChIP-seq datasets were obtained using 14 samples from one-year-old children which were also used for H3K4me3 ChIP-seq. The average yield was ~56M single-end, 151 bp reads per sample. Raw reads were mapped to the hg19 genome using bowtie2-2.2.6 (15) with default settings. The data processing pipeline was identical to the processing of H3K4me3 data with the exception that peak calling was performed using MACS2-2.1.1.20160309 (19) with an input dataset for control and the arguments --broad --broad-cutoff 0.01. Approximately 34,000 H3K27ac peaks were identified for each dataset. DESeq2 was implemented as described above and using default normalization and with delta HAZ scores in DESeq2 design.

Estimation of H3K4me3 ChIP-seq Background and Mistargeted Signal

Proper normalization is a critical component of ChIP-seq data analysis and currently remains an area of active investigation (22-27). We acquired the H3K4me3 ChIP-seq datasets whose analysis is presented here beginning in ~2013, as concerns were emerging (initially from gene expression analysis) that typical "median-based normalization methods would likely miss global effects on expression level (28). As shown in Fig. 2, a global reduction in H3K4me3 signal was observed at "canonical" peaks proximal to TSSs. For datasets with comparable total read numbers, then, we expected the read numbers in regions outside these peaks to be increased in stunted compared to control children. If non-peak reads exclusively represented background, then given that the amount of non-specific ChIP DNA extracted from each cell of each sample should be comparable regardless of sample type (i.e., stunted or control), the true background levels should be the same and hence could serve as a good normalization factor for each H3K4me3 sample. However, as described in the main text, the comparable total levels of H3K4me3 across samples (determined by Western blotting) and the redistribution of H3K4me3 from TSS regions to other regions of the genome in stunted children strongly suggested that the increased signal in non-peak regions represents a mixture of background and mainly non-targeted H3K4me3 signal. If this were the case, then low read count "background" should not necessarily be comparable. To assess whether the low read count data was pure background and hence could be used to normalize samples, or mixed background and low-lying mistargeted H3K4me3 signal, we first performed a rigorous analysis of low read count data.

After exploration of different background models including the Negative Binomial and Poisson distributions, we found that a simple exponential background model outperformed the Negative Binomial and Poisson models and fit the low read count or "background" reads extremely well (Fig. 2D,E). Notably, this was also the case for ENCODE H3K4me3 data obtained from human PBMCs (Fig. S2A). More specifically, we modeled the number of bins with k background reads, $B(k)$, as an

exponential model $B(k) = B(0)e^{-\lambda k}$ where $B(0)$ and λ are the number of bins with no reads and exponential decay parameter, respectively. As is common with ChIP-seq data (regardless of the background model used), we found that the observed number of bins with no reads was in some cases much larger (inflated) compared to a value of $B(0)$ consistent with an exponential model fit with the rest of the background data. Consequently, we fit the natural log of the number of bins with k background reads versus k for background bins containing at least one read (i.e., $k \geq 1$) with the following linear model,

$$\ln B(k) = \ln B(0) - \lambda k,$$

which fit the background data well (Fig. 2D,E). With estimates of the parameters from the fit to background data, $\widehat{B(0)}$ and $\hat{\lambda}$, we summed the number of bins with k background reads multiplied by the number of reads per bin (k) over all k (incurring a negligible error by summing to infinity) to arrive at an estimate of the total background read count,

$$B_T = \sum_{k=0}^{\infty} \widehat{B(0)}\, e^{-\hat{\lambda} k} k = \frac{\widehat{B(0)} e^{-\hat{\lambda}}}{\left(1 - e^{-\hat{\lambda}}\right)^2}.$$

Because background read values tended to be in the tens of millions, we divided B_T by ten million (10^7) to arrive at the total background-based normalization, $N_{B_T} = \frac{B_T}{10^7}$. We were then able to arrive at estimates of the scaled (or normalized) signal, S, for each sample by first subtracting B_T from the total read count for every sample, T, and then dividing that difference by N_{B_T}, $S = \frac{T - B_T}{N_{B_T}}$. For the H3K4me3 differential enrichment analysis using DESeq and correlation analysis of biomarkers and H3K4me3 peaks across samples, we normalized each sample by dividing read counts in peak regions by N_{B_T}. Notably, we performed an experiment in order to determine whether using N_{B_T} or DESeq2's default normalization compared more favorably to the normalization suggested by chromatin spike-in read counts. The analysis discussed below details how we found that DESeq2's default normalization matched that of the spike-in read counts much better than N_{B_T}.

Comparison of ChIP-seq normalization methods

The results reported in the main text utilized DESeq2 default normalization. After these datasets had already been generated and concerns about global changes in modification became more apparent, much like the strategy used for global normalization of RNA-seq data (28), spike-in chromatin was developed to test for global changes in signal that would otherwise be masked (24). Thus, to test in an alternative way for a global change in H3K4me3 levels in stunted compared to control children, and to assess our alternative normalization approaches, we generated four datasets with spike-in reads post hoc. These consisted of two datasets from control and two from stunted children. Using these 4 datasets, comparative analyses were run with three types of normalization: (1) DESeq2 default normalization, (2) spike-in read count-defined normalization and (3) normalization based on the background model described in the previous section. Normalization factors based on method (2) were obtained by dividing the spike-in read counts by ten million. As shown in Table S2, we observed notable consistency in the normalization factors obtained using spike-in read counts and the DESeq2 default normalization method, in strong contrast to the normalization factors obtained by

considering non-peak reads as background only and not representing background plus true, mistargeted, H3K4me3 signal.

Differential peak analysis using DESeq2 in combination with each of the three sets of normalization factors indicated that DESeq2 default normalization and spike-in normalization gave rise to very similar patterns of differentially affected peaks when comparing the two stunted datasets to the two control datasets (Fig. S2C and E). In contrast, normalization using the background model normalization factors showed a very different pattern in which the majority of peaks were strongly decreased in stunted compared to control children, as would be expected if the increased H3K4me3 signal in non-peak regions were considered as background and normalized away. Moreover, there was broad overlap in sets of significantly affected peaks identified using DESeq2 default normalization and spike-in normalization methods, with the differentially affected peaks from spike-in normalization nearly entirely contained within the set of differential peaks identified using DESeq2 default normalization (Fig. S2F). Consistent with the broad overlap in the differentially affected peak sets using these two normalization methods, we found that the genes associated with the two sets of differential peaks were enriched in similar sets of gene ontology terms, including terms related to immune system (dys)function and transcription as we report for the full set of 52 week data (Fig. S3). We conclude from this analysis that DESeq2 default normalization and spike-in normalization identify very similar sets of affected peaks and associated with very similar underlying biology.

<u>Differential peak analysis and functional annotation</u>

Our original set of H3K4me3 data consisted of 24 datasets from children at 18 weeks of age and 21 datasets from children at 52 weeks of age. Two datasets from each of these age groups were set aside as they had quantile read distributions indicative of library over-amplification. An additional 18-week dataset was attributed to a male but had the epigenetic signature of a female, indicating a probable sample mix-up at some point between the blood draw and archiving of the resulting PBMC sample. This yielded 21 datasets from 18-week children that were used in the analysis. Similarly, two of the original datasets from children at 52 weeks of age were of poor quality (read distributions reminiscent of input samples) and were set aside. The available clinical data suggest that stunting has multiple contributing factors that can be associated with more than one pathway for disease emergence (29). Indeed, we saw by Principal Component Analysis that 3/21 datasets (one control and two stunted samples) did not group with phenotypically similar samples (not shown). It is likely that these three datasets represent the patterns in either children with other disease conditions or children having arrived at the same overt presentation via a different route(s). These three outlier datasets were set aside, and the analyses presented in the main text were obtained from 16 52-week samples (~76% of the original data) and as described in the main text. All 24 maternal datasets were used in the analysis of maternal H3K4me3.

Based on the background and spike-in analyses described above, differential peak analysis of 18-week, 52-week and maternal data was performed in R using DESeq2 and employing the DESeq2 default normalization method (21). We compared samples to obtain differentially affected peaks based on phenotypic differences (e.g. control versus stunted) in which each sample of a given type was treated essentially as a replicate. A more powerful approach was to use various biomarkers including HAZ score and delta HAZ score as quantitative measures of the degree of stunting. In this type of DESeq2 analysis, significantly affected peaks were identified as those with statistically significant changes correlating with the biomarker value across all samples. In these cases, the log2FC values represent the incremental change in peak size per unit of the particular biomarker. Significant

differential peaks were defined as those with FDR-adjusted p-values < 0.05. PCA analysis was performed in R using prcomp with regularized log transformation of the DESeq2 results as input. The results presented here for the children were obtained using both male and female datasets together. As shown in Fig. 3A, males and females were readily distinguishable by PCA. Analyses were also conducted using only the autosomal peaks, and of females alone and males alone. Leaving out peaks on the sex chromosomes had very little impact on the results overall and provided no insight into possible sex differences in stunting-associated autosomal peaks. In the analysis of datasets by sex, fewer significantly affected peaks were identified, most likely due to the reduced statistical power associated with the relatively small number of datasets used in the analyses of only one sex at a time.

Significantly affected peaks were computationally associated with genes using GREAT (30), selecting the whole hg19 genome as the background region and with default gene association settings. Transcription start site histograms were generated using the distance of the peak to the nearest TSS as defined by GREAT. GREAT provided gene set enrichment results as well; although the gene set enrichment results from GREAT are not reported here, they were broadly consistent with those obtained using other enrichment tools. To define the possible functional significance of H3K4me3 peak changes, genes from GREAT were re-associated with the DESeq2 peak log2FC and/or adjusted p-values in python in order to obtain ranked gene lists, which were used for functional enrichment analysis in DAVID (31), GOrilla (32), MSigDB and GSEA (33), and Pathway Express (34). For each gene set enrichment tool, we submitted gene lists of the recommended maximum size and analyzed genes associated with peaks with positive log2FC values separately from genes associated with peaks with negative log2FC values. As described in the main text, when analyzed with respect to delta HAZ, the peaks with positive log2FC values were overwhelmingly in stereotypical locations in or around transcription start sites whereas the peaks with negative log2FC associations with delta HAZ were rarely if ever proximal to TSSs. For this reason, we focused mainly on understanding the functional significance of genes with positive log2FC peaks as they likely are transcribed at rates that scale with the health of the child. In general, functional analysis of gene sets explored using each tool above resulted in broadly similar sets of results.

Pathway enrichment and upstream regulator results shown in Figs. 4A, B and C were obtained using Ingenuity® Pathway Analysis (IPA®, QIAGEN Redwood City, www.qiagen.com/ingenuity). As explained above and in the main text, for analysis of data from one-year-old children, we focused on genes associated with peaks showing positive log2FC values versus delta HAZ score. However, very similar Canonical Pathway enrichment results were obtained by analyzing genes associated with differential peaks located within 1.5 kb of the TSS (includes those with both positive and the few with negative log2FC values) as well as genes associated with peaks that decreased in the categorical comparison of stunted versus control children. Analysis of maternal data focused on all peaks associated with maternal height. For analysis of the one-year old data, a list of the top 3000 genes with positive log2FC values ranked by DESeq2-derived adjusted p-value was used as input. Significantly affected pathways were defined as those with an IPA p-value of < 0.05. We have focused on gene ontology and pathway enrichment related to protein coding genes, but our analysis uncovered many links to miRNAs and other regulatory molecules which were not explored further.

Gene or peak average plots were generated using ngs.plot (35). In addition to settings to specify rendering for publication (font size, line width, etc), -MW 9 was used.

Enhancer analysis

To determine the relationship between enhancers and delta HAZ affected H3K4me3 peaks in one year old children, BEDTools was used to identify peaks that overlap by one base pair or more with

enhancers that are active in various cell types as reported previously (36). "Positive peaks", those that increase with increasing delta HAZ score, and "negative peaks", those that decrease with increasing delta HAZ score, were analyzed separately. The peak-enhancer overlap results are summarized in Table S2. Genes targeted by the subset of enhancers with overlapping differential H3K4me3 peaks were identified from the list of enhancer-gene associations reported in the same study that identified the catalog of active enhancers (36).

Motif analysis

The DNA sequences of the enhancers identified above were retrieved from the UCSC repository (http://genome.ucsc.edu/) (37). Enriched DNA sequence motifs in these enhancers were then identified using MEME (http://meme-suite.org)(38). The total enhancer sequence length was far too great to search in its entirely, so subsets of randomly selected enhancer sequences were chosen using the MEME Suite tool fasta-subsample, resulting in 142 and 155 enhancers associated with H3K4me3 peaks that increased ("positive peaks") or decreased ("negative peaks"), respectively, with delta HAZ score. The subsample sequence numbers were determined empirically to result in close to the maximum input file sizes for analysis by MEME. MEME v4.11.2 was run using -maxsize 60000 -mod zoops -nmotifs 3 -minw6 -maxw 50 -revcomp and appropriate background files. Background files were generated using fasta-get-markov with -m 3. Significantly enriched motifs were associated with known transcription factor motifs using the MEME Suite tool TOMTOM with the HOCOMOCO Human v10 database.

For the analysis of enhancers associated with negative peaks, the two highest scoring motifs are shown in Fig. 3F. The ETS1-associated motif (155 sites in 155 enhancers) was discovered with a TOMTOM E-value of 2.12×10^{-3} whereas the FOXO1-associated motif (47 sites in 155 enhancers) was discovered with an E-value of 3.40×10^{-3}. The third motif (not shown) had no statistically significant association with known motifs in TOMTOM. The enhancers associated with positive peaks included an A-rich motif (44 sites in 142 enhancers; E-value: 2.0×10^{-57}) and a C-rich motif (87 sites in 142 enhancers; E-value: 1.3×10^{-46}). Much like the A-rich motif discovered for the negative peaks, the A-rich motif identified with the positive peaks was associated with FOX-family transcription factor motifs in TOMTOM (E-value for FOXJ3, the top scoring motif: 3.29×10^{-2}). The C-rich motif was found using TOMTOM to be similar to MAZ, EGR1, and Sp family transcription factor motifs, among others, with the E-value for the top scoring MAZ association 1.56×10^{-6}. The third motif identified using MEME associated with the positive peaks had no significant association with known transcription factor motifs using TOMTOM.

H3K4me3 peak-adult height snp overlap analysis

To determine the relationship between all H3K4me3 peaks associated with delta HAZ score at one year of age and single nucleotide polymorphisms (snps) associated with adult height, BEDTools was used to count peaks that overlapped by one base pair or more with 602 snps linked to adult height and reported previously (39). The results are summarized in Table S4. As H3K4me3 peak size at the TSS is generally correlated with transcription, those overlapping snps near the TSS may be eQTLs.

Correlation analysis of 18-week, 52-week and maternal datasets

Global correlation analysis shown in Fig. 4E was performed using a collection of nine IDs for which we obtained 18-week, 52-week and maternal datasets for each. Raw counts for all autosomal peaks for each dataset were divided by DESeq2-defined normalization factors, and Pearson

correlations were computed for all pairwise combinations of the datasets. The matrix of correlations was visualized using the heatmap.2 function in R with color specified by the viridis library.

Re-analysis of published H3K4me3 ChIP-seq datasets

H3K4me3 datasets acquired from Hct116 cells grown in the presence or absence of methionine (40) were retrieved from the Gene Expression Omnibus (accession number GSE72131) and analyzed essentially as described above. Raw sequence reads were mapped to the hg19 genome using Bowtie2, the resulting SAM files were converted to BAM format using SAMTools and peaks were called using MACS with input sequences as the control. Reads were distributed to the union set of all called peaks and differential peak analysis was performed using DESeq2, with significantly affected peaks (FDR adjusted p-values < 0.05) associated with genes using GREAT as described above.

H3K4me3 heatmap

The normalized counts for the significant differential peaks (FDR-adjusted p-value < 0.05) were used with the heatmap.2 function in R and with the scale = "row" argument and z-score normalization of peaks across samples. The default row (peak) clustering method was used and samples (columns) were ordered by delta HAZ score.

Roadmap Epigenomics Project Data

Publicly available Roadmap Epigenomics Project ChIP-seq datasets for H3K4me3 and H3K27ac in PBMCs from male and female individuals were obtained from the ENCODE site, reference epigenome series ENCSR841LMD.

RNA-seq data analysis

Raw datasets in FASTQ format were evaluated for quality and uniformity using FASTQC (https://www.bioinformatics.babraham.ac.uk/projects/fastqc/). Files for each sample from each of the four NextSeq500 flow cells were then merged and paired-end reads were mapped to a human hg19-ERCC reference genome using HISAT2 (41). The resulting SAM files were converted to BAM format using samtools (14), and transcripts were assembled and quantified with StringTie (42) using combined GENCODE (gencode.v2lift37)-ERCC annotations. Gene- and and transcript-level count tables were generated using prepDE.py (http://ccb.jhu.edu/software/stringtie/index.shtml?t=manual). Results presented here were obtained using the transcript-level count table. Differential analysis of RNA expression was performed using DESeq2 as described above with delta HAZ as a quantitative variable and following filtering to remove transcripts in which four or more samples had zero reads. Other analyses were performed as described above.

Power, sample size, randomization and exclusion criteria in mouse studies

To estimate the sample numbers required for comparison of control and LRP- mice, we first calculated the mean and standard deviations of the weight-for-age z-scores at one year of age for the control and stunted children whose samples were used for H3K4me3 analysis. These values were used in two-sample tests (43) to estimate sample sizes required to detect a difference in weight associated with loss of LRP1 in mice. This analysis showed that four mice in each group would be sufficient for detection of a difference with alpha = 0.05 and power = 0.99 and is a conservative underestimate of statistical power as the children showed modest differences in LRP1 expression whereas the gene was deleted in knock-out mice.

Runts, mice that weighed significantly less than their sex-matched littermates (prior to tamoxifen injection), were excluded from experiments. Mice were randomly assigned to control and tamoxifen treatment groups, and the mouse experiments were performed without blinding. Tests for data distribution were performed using Prism 6 (GraphPad) software and statistical tests were chosen in part based on the distribution test results. The variance was typically greater in the LRP- mice than the LRP+ mice.

R and other software

Differential ChIP-seq peak analysis was performed in R (v3.3.1) and employed the Biobase_2.32.0, DESeq2_1.12.4, BiocGenerics_0.18.0, genefilter_1.54.2, GenomicRanges_1.24.2, IRanges_2.6.1, and SummarizedExperiment_1.2.3 packages. RNA-seq data analysis was performed using HISAT2 v2.0.4 and StringTie v1.3.4d. Plots were mainly generated using ggplot2_2.1.0, but some images were generated using gplots_3.0.1 and/or lattice_0.20-34 along with RColorBrewer_1.1-2. Venn diagrams were generated using Biovenn (44) and then edited for presentation using Adobe Illustrator CS6. Statistical results not derived from a particular tool or package described above were obtained in R using t.test() or fisher.test().

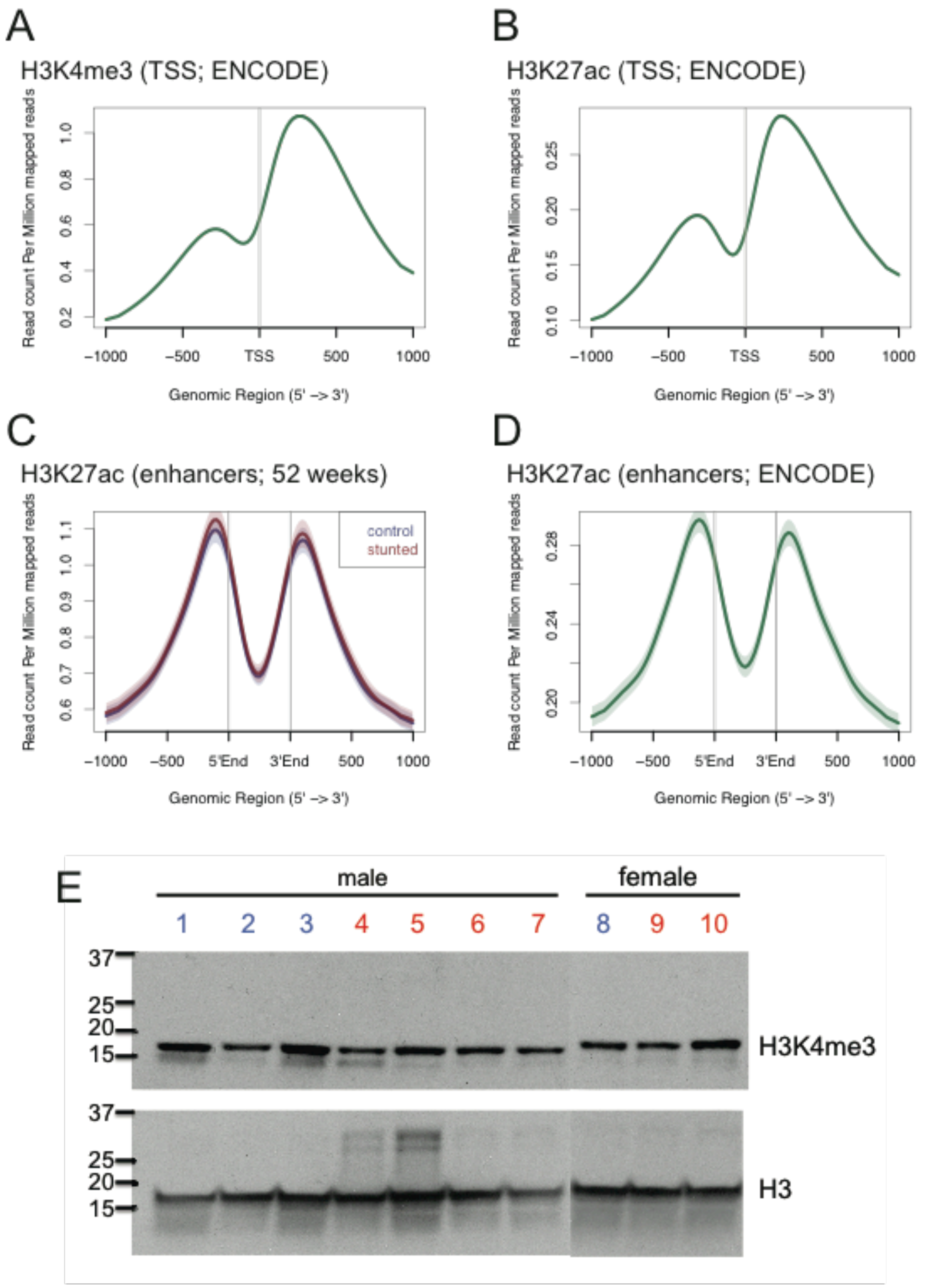

Fig. S1. A, Gene average H3K4me3 profile at transcription start sites (TSSs) in PBMCs as measured by the Epigenome Roadmap Project and available through ENCODE (4, 45). **B**, Gene average H3K27ac profile at TSSs in PBMC data reported by ENCODE. We expect the genome-wide distributions of H3K4me3 and H3K27ac to be correlated (46), and indeed, the Pearson correlation coefficients between these two marks in datasets from children at 52 weeks of age ranged from 0.722

to 0.825, with a median value of 0.785 and comparable to the correlation in ENCODE data of 0.846. Moreover, there was no decay in correlation value with stunting. Collectively, these results reinforce the conclusion that the H3K4me3 data associated with stunted individuals is of high quality. **C**, H3K27ac average profiles at blood cell enhancer regions (36) in PBMCs from control and stunted children at 52 weeks or (**D**) as reported by ENCODE. The results in (**C**) and (**D**) show that H3K27ac in control and stunted children is localized at active enhancers as expected (47). **E**, Representative western blots of total histone H3 and total H3K4me3 in chromatin samples from one-year-old children. Each lane was loaded with 5 mg of a different sample; samples from controls are labeled in blue, samples from stunted children are labeled in red.

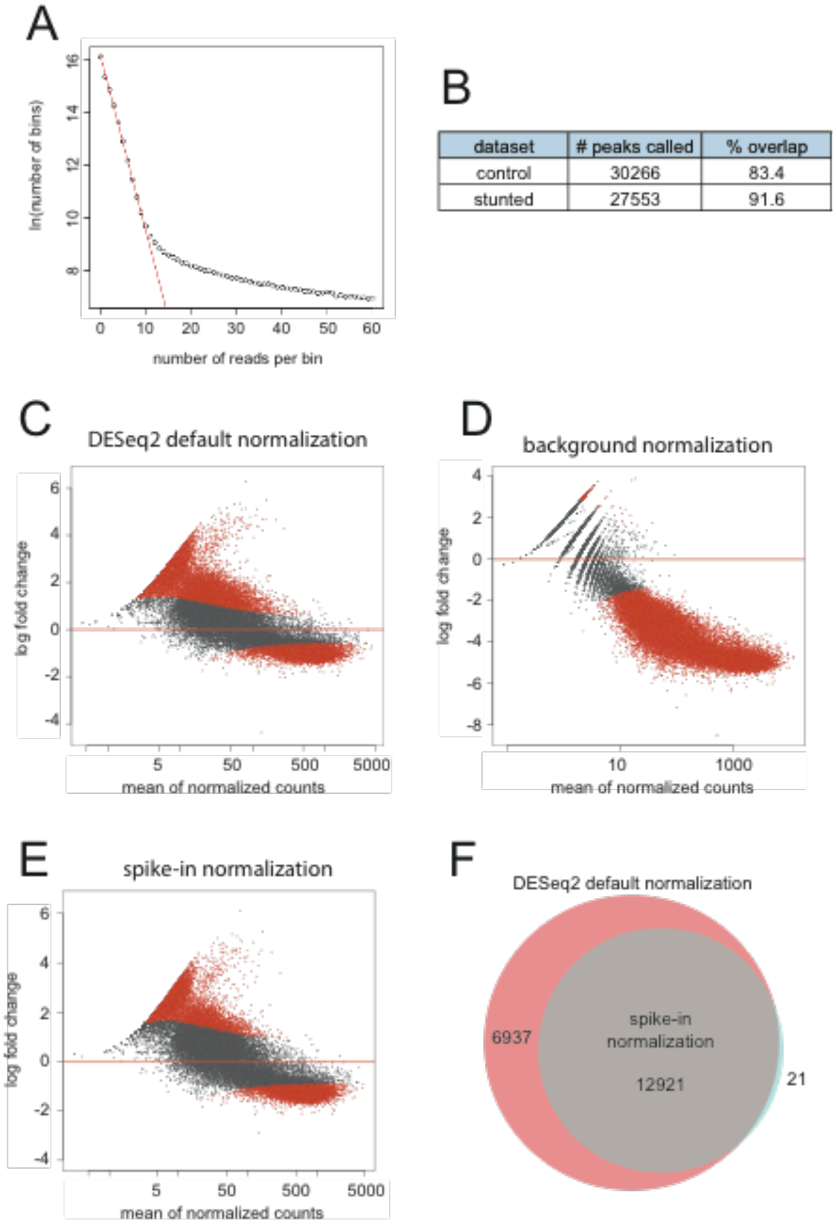

B

dataset	# peaks called	% overlap
control	30266	83.4
stunted	27553	91.6

Fig. S2. **A**, Exponential background model fit of ENCODE H3K4me3 data obtained from human PBMCs. Compared to the H3K4me3 datasets presented here (Figs. 2E,F), the ENCODE data show differences in the distribution of reads in peaks versus background as would be expected given differences in sample preparation, sequencing platform and sequence read depth. Nonetheless, the exponential background model fits the ENCODE data comparably well. **B**, Numbers of H3K4me3

peaks called in datasets obtained from stunted or control children at one year of age. The high degree of overlap indicates that there was high concordance in the number and location of H3K4me3 peaks despite the overall reduced level of signal in peaks in stunted datasets. **C-E**, Comparison of differential peak calls (stunted versus control children at one year of age) using three different normalization methods. This analysis was performed using the 4 datasets described in SI Methods. The plots show the mean normalized read counts versus log2 fold change obtained after read count normalization by the method indicated in the plot title. Red denotes FDR-corrected p-value < 0.05. Note the similarity in the results obtained with DESeq2 default normalization and spike-in normalization. **F**, Overlap (gray) in differential peaks identified using DESeq2 default normalization (pink) or spike-in normalization (cyan). The results were obtained using the 4 spike-in datasets described in SI Methods. 99.84% of the significant (FDR-corrected p-value < 0.05) differential peaks identified using spike-in normalization were identified using DESeq2 default normalization.

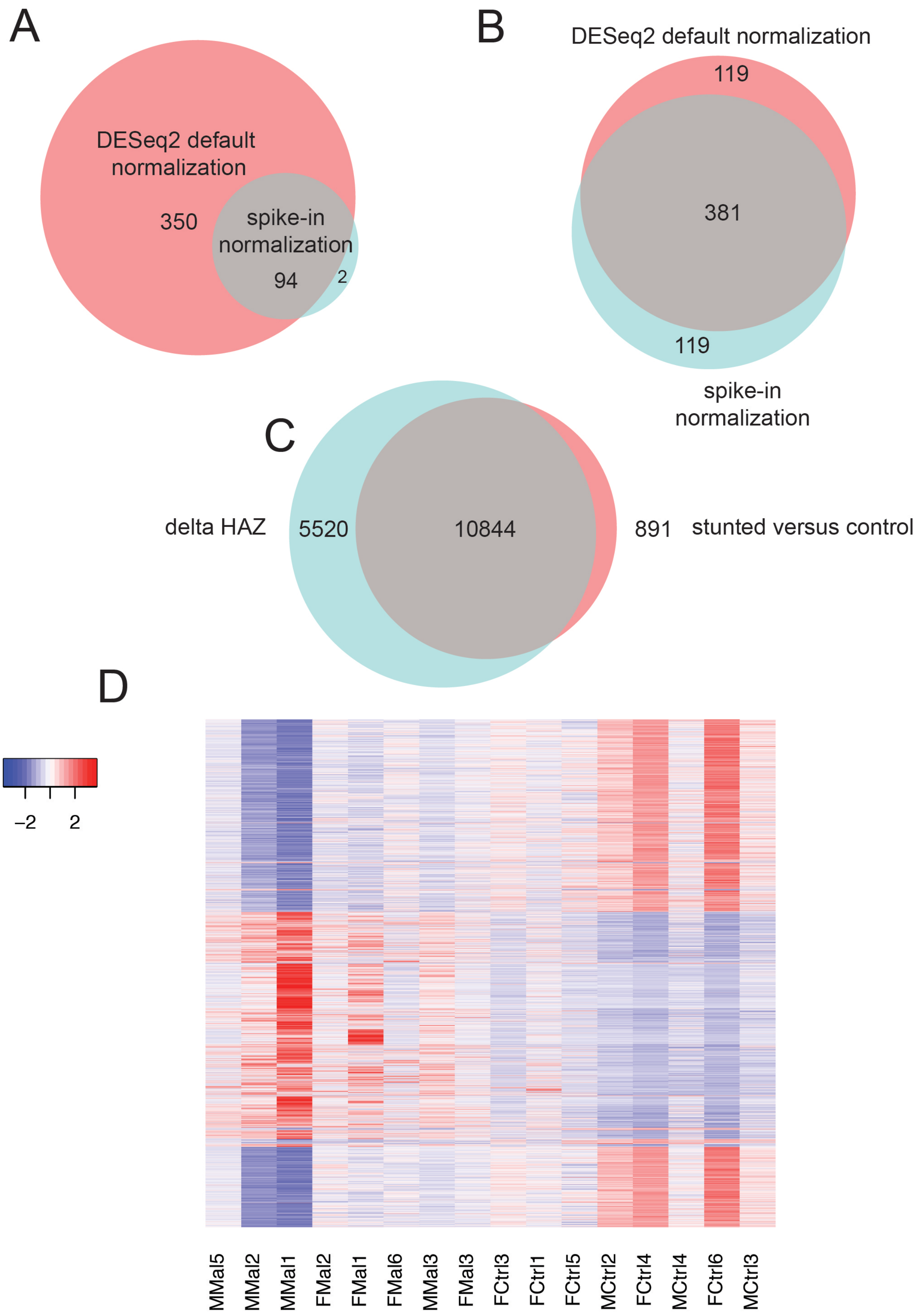
A
DESeq2 default normalization
350
spike-in normalization
94
2
B
DESeq2 default normalization
119
381
119
spike-in normalization
C
delta HAZ
5520
10844
891
stunted versus control
D
−2
2
MMal5
MMal2
MMal1
FMal2
FMal1
FMal6
MMal3
FMal3
FCtrl3
FCtrl1
FCtrl5
MCtrl2
FCtrl4
MCtrl4
FCtrl6
MCtrl3

Fig. S3. (**A**) and (**B)** show overlaps in MSigDB Canonical Pathway Terms associated with differentially affected H3K4me3 peaks identified using DESeq2 default normalization or spike-in normalization. Differentially affected peaks were computationally associated with genes, then the gene lists were tested for functional enrichment using MSigDB as described in Methods and Supplementary Information. **A**, Overlap in Canonical Pathway terms with binomial FDR Q values < 0.05. DESeq2 default normalization captured 97.8% of the enriched terms identified using spike-in normalization, plus other terms. **B**, Since the analysis only included 4 datasets and was therefore likely underpowered for defining functional enrichment, also shown is the overlap in all functional terms identified regardless of FDR Q value. The figure shows that the overlap in overall Canonical Pathway Terms was 76.2%. In comparison to the total number of Canonical Pathway Terms in the MSigDB database, overlaps in both A and B were found to be highly significant ($p < 2.2 \times 10^{-16}$ using Fisher's Exact Test). **C**, Overlap in differential H3K4me3 peaks identified in a comparison of datasets from control and stunted children at one year of age versus those peaks with significant log2 fold-change per unit of the child's delta HAZ score. Differential peak analysis using delta HAZ score captured 93.4% of the peaks identified in the categorical comparison of control and stunted children, and in addition revealed 5520 additional peaks associated with growth within the first year of life. **D,** The heatmap shows the 16,477 peaks (rows) with significant H3K4me3 differences versus delta HAZ score at one year of age. Samples (columns) are ordered from lowest (left) to highest (right) delta HAZ score. Control samples are labeled Ctrl and stunted samples (HAZ score < -2) are labeled Mal. The first letter M or F in each sample name designates male or female, respectively. Counts in each peak were z-score normalized and peaks were clustered using default settings in R using heatmap.2. Row names were omitted for clarity.

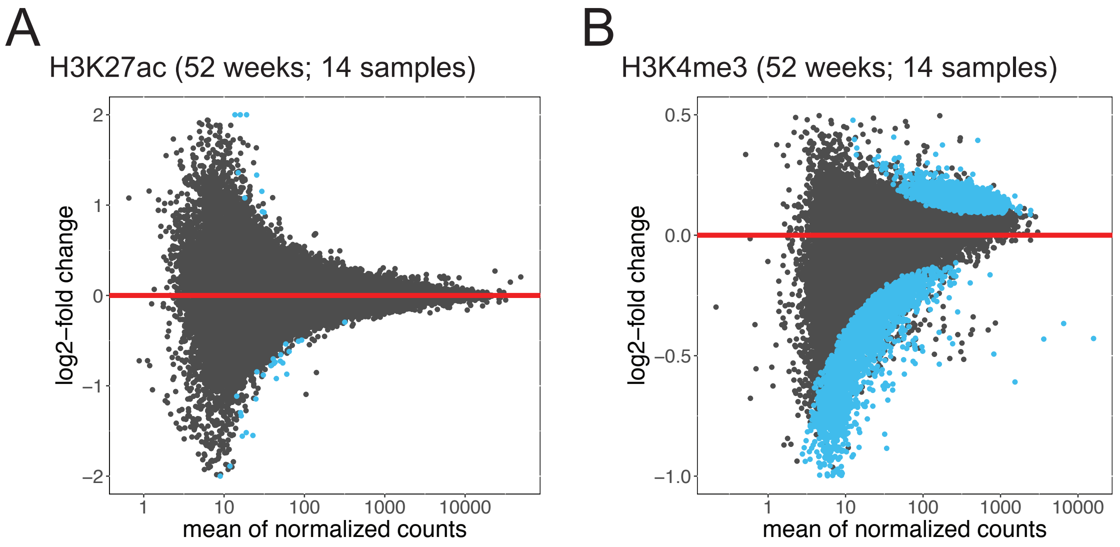

Fig. S4. **A**, Plot of H3K27ac peak size versus log2-fold change with respect to delta HAZ score in 14 children at one year of age. **B**, Plot as in (**A**) for H3K4me3 peaks. H3K27ac was measured using all chromatin samples from 52-week-old children for which sufficient material was available after measuring H3K4me3. The plot in (**B**) was derived from datasets from the same 14 samples for which H3K27ac was measured in (**A**) and so provides a direct comparison of the significant peak changes for the two marks in the very same samples. For comparison, the H3K4me3 results shown in Fig. 3B were obtained using all 16 datasets from 52-week-old children. In both (**A**) and (**B**), peaks with log2-fold change values associated with FDR-corrected p-values < 0.05 are colored in cyan.

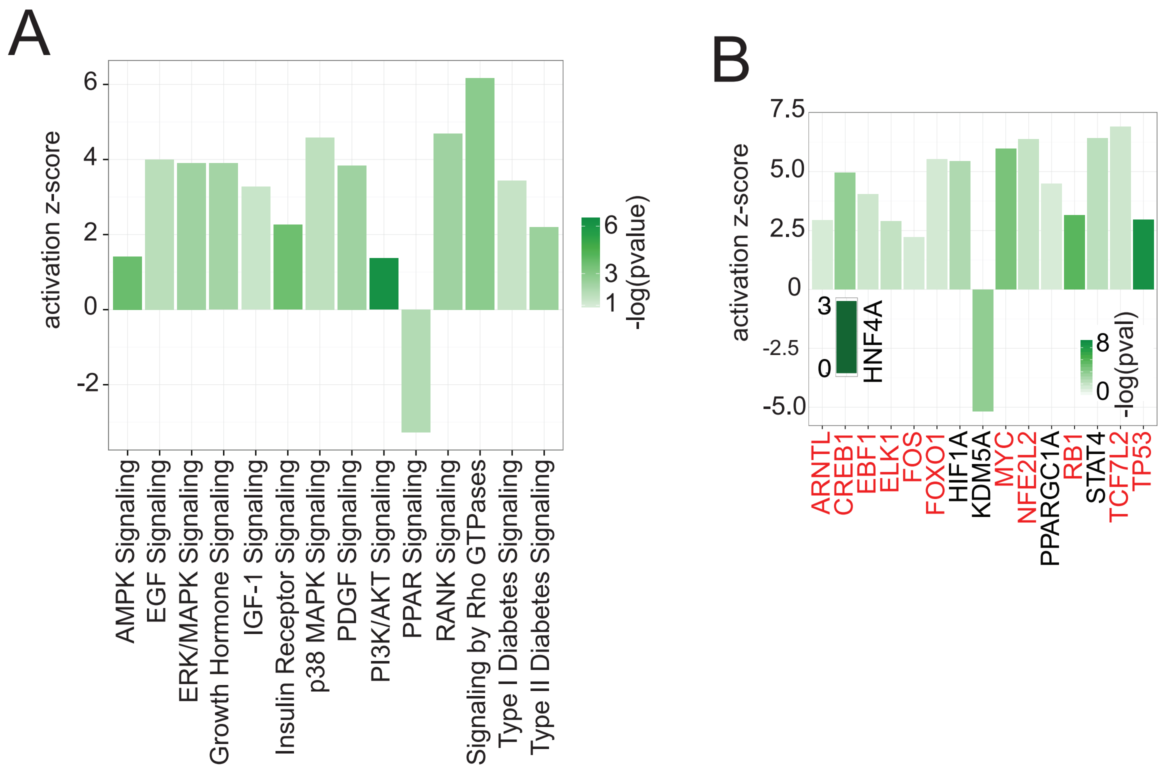

Fig. S5. A, Changes in growth and proliferation pathways in one-year-old children based on differential H3K4me3 peaks associated with growth trajectory (delta HAZ score) using Ingenuity Pathway Analysis. Positive z-scores indicate predicted pathway activation with increasing overall health; negative z-scores indicate predicted pathway activation with stunting. Note that all but one of these pathways had positive activation z-scores, meaning that the pathways were predicted to be more activated in healthy compared to stunted children as would be expected. **B**, Differentially affected H3K4me3 peaks are associated with genes whose expression is driven by the indicated transcription factors (TFs). Positive z-scores indicate an increasing likelihood that the TF is activated with increasing health; the negative z-score for KDM5A indicates that KDM5A is increasingly inhibited with increasing health, thus, increased KDM5A (H3K4me3 demethylase) activity in stunted children. Inset: HNF4 had a –log(p-value) = 31.05. The list also includes general regulators of cellular growth (MYC, RB1, TP53), regulators of metabolism and stress in blood cells (TCF7L2, NFE2L2, HIF1A), and regulators of immune cell growth and development (FOS, ARNTL, EBF1, STAT4). NFKB subunit genes were also associated with differentially affected H3K4me3 peaks, providing support for the association of altered inflammatory responses with stunting. FOXO1 was also identified in this analysis as a key transcription factor, supporting the observation above that FOXO1-targeted enhancers are specifically affected in stunted children. Since AKT-mediated phosphorylation of FOXO1 regulates chromatin occupancy and transcriptional activity (48), reduced TOR and AKT signaling in stunted children (A) would further potentiate FOXO1 misregulation.

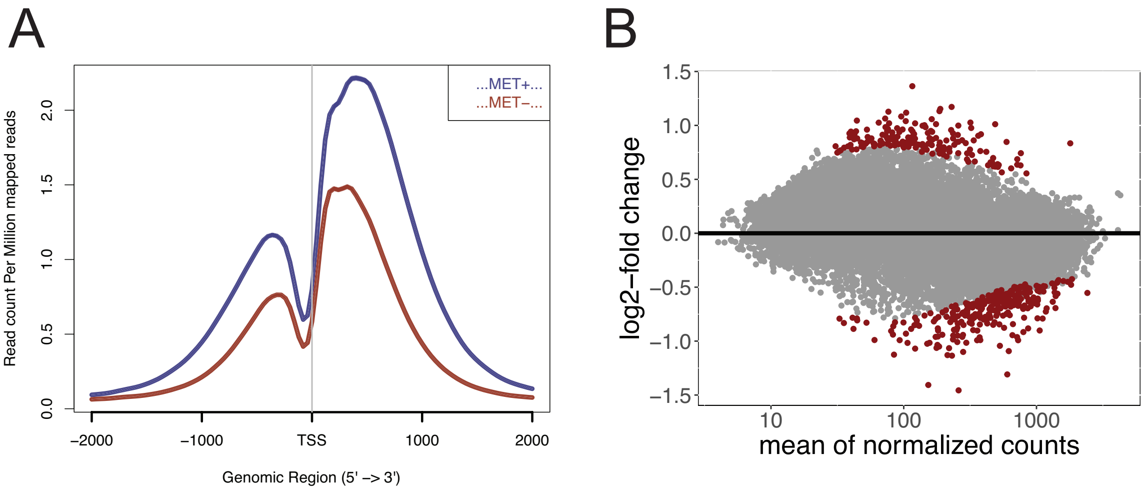

Fig. S6. A, Gene average plots of histone H3K4me3 levels with respect to transcription start sites (TSS) in Hct 116 cells grown in the presence (blue) or absence (red) of methionine added to the culture medium. **B**, Normalized mean H3K4me3 signal is plotted versus the log2 fold-change in cells grown in minus methionine versus plus methionine conditions. Each dot represents a peak; red dots have FDR-corrected p-values < 0.05. Results in **A** and **B** were obtained by re-analysis of ChIP-seq data reported in (40).

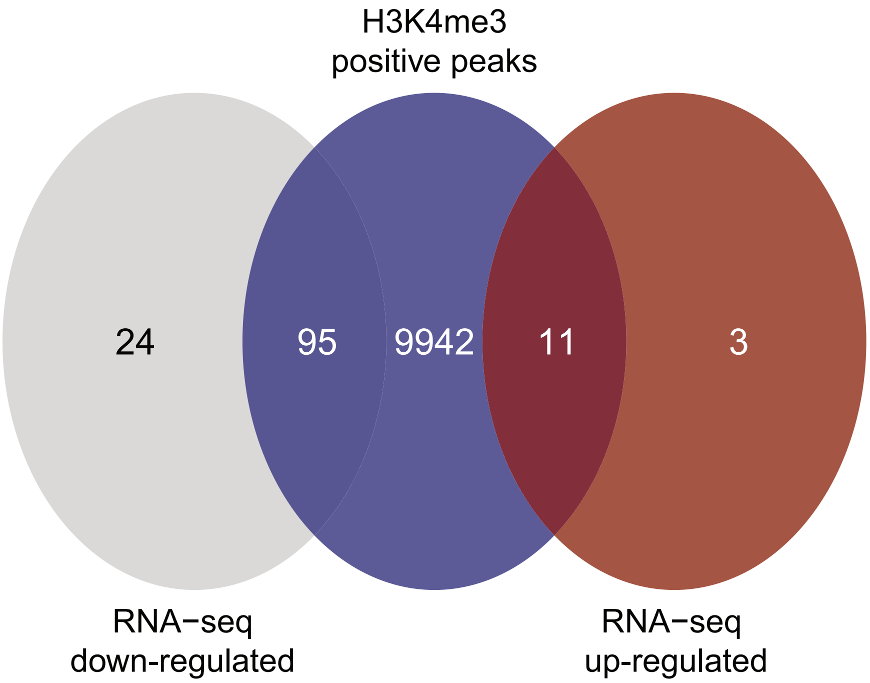

Fig. S7. Venn diagram showing the overlap in the genes associated with H3K4me3 positive peaks (significantly affected peaks that increase with health) and significantly affected genes identified by RNA-seq. Up-regulated genes were those whose expression increased with health; down-regulated genes were those whose expression decreased with health. Both overlaps are highly significant: $p < 1.71e-9$ for the up-regulated genes overlap and $p < 3.08e-11$ for the down-regulated genes overlap.

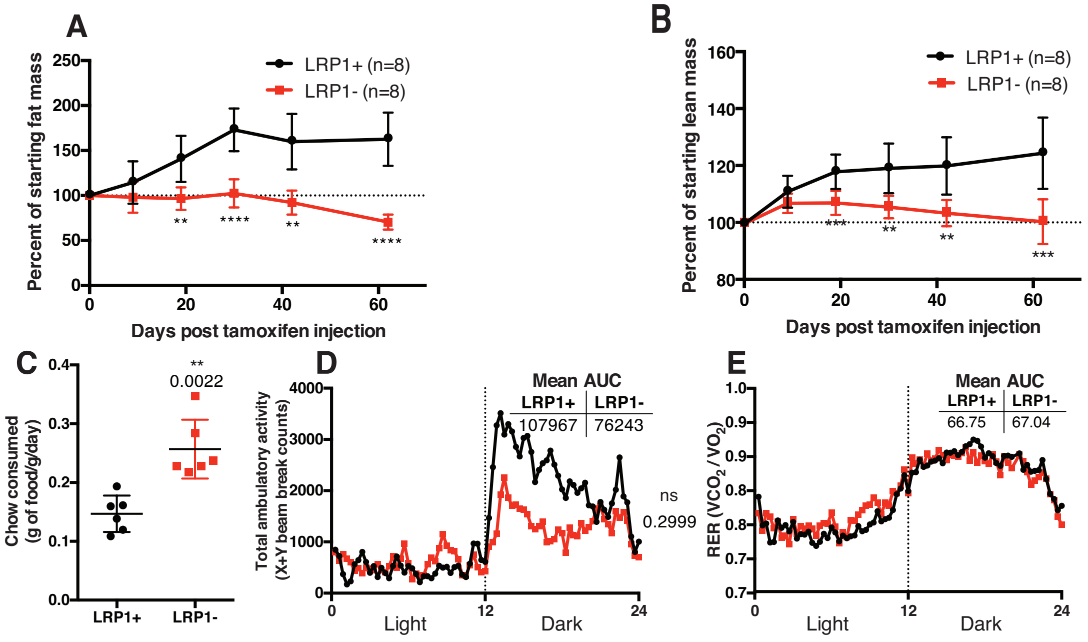

Fig. S8. **A**, Percentage weight gain in LRP-1$^{fl/fl}$Cre-ERt2^{+} (LRP1-) and LRP-1$^{+/+}$Cre-ERt2^{+} (LRP1+) mice after injection of tamoxifen. The plot is similar to Fig. 5B but the data are expressed as the percent of starting fat mass. **B**, Plot as in **A** but data are expressed as the percent of lean mass. **C**, Plot of chow consumed in LRP+ and LRP- mice. Each dot is an animal. **D**, Total ambulatory activity in LRP1+ (black) and LRP- (red) mice (N=4). The x-axis scale is in hours. The trend in somewhat reduced activity in LRP1- mice compared to control mice was not significant. **E,** Plot of respiratory exchange ratio (RER) in LRP1+ (black) and LRP1- (red) mice. The x-axis scale is in hours. The results in this figure as well as those in Fig. 5 have both similarities and differences to reported observations of mice with tissue-specific knock-outs of LRP1 (49) (50); to date, no single tissue knock-out of LRP1 has resulted in the full range of phenotypes reported here.

Table S1.

H3K4me3 and H3K27ac ChIP-seq datasets.

Child dbGaP Subject ID	H3K4me3 dataset for child at 18 weeks	H3K4me3 dataset for child at 53 weeks	H3K27ac dataset for child at 53 weeks	Maternal dbGaP Subject ID	H3K4me3 Maternal dataset	Famil y ID	child sex	HAZ score (week 53)	delta HAZ score (week 52)	child phenotype at one year of age
db0924		√	√			2	f	-0.68	-0.95	control
db2811		√	√			4	f	-3.2	-1.17	stunted
db0603		√	√			5	m	-3.68	-2.78	stunted
db1925		√	√			6	m	-3.71	-2.05	stunted
db3572		√	√			7	f	-1.45	-0.48	control
db2571		√	√			8	f	-3.24	-1.53	stunted
db2757		√				9	f	0.8	1.67	control
db7669		√	√			10	f	-2.67	-1.13	stunted
db2939	√			db5120	√	11	f	-2.97	-1.55	stunted
db9649	√			db3968	√	13	f	-3.13	-0.98	stunted
db6560		√	√			14	m	-3.01	-3.13	stunted
db7007	√	√				15	m	0.61	0.68	control
db8613	√			db4973	√	16	m	-4.06	-2.74	stunted
db5113		√	√	db2800	√	16	f	0.56	0.02	control
db4462		√	√	db1172	√	17	m	-4.25	-1.4	stunted
db8078	√	√	√	db5558	√	18	m	1.6	1.19	control
db6596	√			db9097	√	19	f	0.22	0.74	control
db5574	√					20	m	0.03	-0.38	control
db6557	√			db2145	√	21	f	-2.5	-2.56	stunted
db5121		√	√			22	f	-3.08	-1.65	stunted
db2613	√	√	√			23	f	1.22	0.86	control
db8425		√	√	db1967	√	24	m	0.84	1.32	control
db4113	√			db7241	√	25	f	-0.34	-0.78	control
				db4398	√	26	f	-0.48	-0.07	control
db1903	√			db2875	√	27	f	0.18	0.38	control
db5287	√			db8274	√	28	m	0.33	1.06	control
db9661	√			db2368	√	29	m	-2.28	-2.06	stunted
db5879	√			db1599	√	30	m	-2.6	-0.14	stunted
				db0288	√		m	-0.3	-0.63	control
db3461	√			db6651	√	32	f	-2.78	-1.07	stunted
db0063	√			db6501	√	33	m	-0.16	-0.69	control
				db4694	√		m	0.21	-0.44	control

db6561	√			db2922	√	34	f	-0.56	-0.25	control
db0840	√			db773	√	35	f	-3.18	-1.29	stunted
db8903	√			db8636	√	36	m	-2.26	-0.89	stunted
db1709	√			db6358	√	37	f	-2.97	-2.35	stunted
db1120	√			db7005	√	38	m	-2.35	-2.24	stunted

Table S2.
Normalization factors obtained using 3 different methods.

dbGaP Subject ID	age	sex	phenotype	Normalization Factor: DESeq2 default	spike-in	background model
db2757	53 weeks	female	control	2.2389106	1.667487	0.545233117
db7007	53 weeks	male	control	2.2812397	2.592137	0.595571042
db0603	53 weeks	male	stunted	0.3689791	0.285365	2.7817753
db6560	53 weeks	male	stunted	0.5578444	0.806104	1.89690977

Table S3.

Number of active enhancers that overlap with H3K4me3 peaks.

tissue/cell type	# peaks up*
blood	192
T cells	597
monocytes	769
dendritic cells	391
lymph node	6
macrophages	176
natural killer cells	322
neutrophils	238
reticulocytes	61
thymus	33
	2785 total
	1210 unique

*positive log2FC with delta HAZ score

**negative log2FC with delta HAZ score

Table S4.
Overlap between height snps* and delta HAZ associated H3K4me3 peaks in children at one year of age.

snp		peak			delta HAZ		
chr	position	chr	start	end	log2FC**	gene	distance to TSS
chr15	99194896	chr15	99189630	99195961	0.1526	IGF1R	596
chr15	74220599	chr15	74218416	74220921	0.1476	LOXL1	870
chr16	764826	chr16	764402	766263	0.1654	METRN	218
chr17	27917771	chr17	27914749	27917788	0.1071	ANKRD13B	-4217
						GITI	298
chr19	2152018	chr19	2149505	2152392	0.1279	AP3D1	591
chr20	32077916	chr20	32076840	32079368	0.1243	CBFA2T2	-72067
						SNTA1	-46406
chr22	23585132	chr22	23585016	23585846	-0.2178	BCR	63034
						IGLL1	337064
chr3	185541213	chr3	185540471	185544510	0.1033	IGF2BP2	353
chr4	106071064	chr4	106065629	106071314	0.1176	TET2	1022
						PPA2	326766
chr4	57847220	chr4	57841057	57847428	0.1238	POL2R2B	335
						NOA1	746
chr5	131633355	chr5	131627862	131635120	0.1507	SLC22A5	-73953
						SLC22A4	1355
chr6	130341235	chr6	130338940	130342815	0.1629	L3MBTL3	6034
						SAMD3	345692
chr6	5260812	chr6	5259899	5263110	0.1104	LYRM4	-333
						FARS2	228
chr6	34204285	chr6	34202797	34206999	0.1238	HMGA1	248
chr6	80816296	chr6	80815282	80817301	0.1366	BCKDHB	-72
chr7	158649005	chr7	158648242	158650409	0.147	WDR60	57

*Wood et al, Nat. Genet. 46:1173-1186, 2014

**log2FC in peak per unit of delta HAZ; positive values signify peaks that increase with health and vice versa for negative

peak also correlates with maternal height

distance in bp within 2 kb of TSS

Table S5
Differentially expressed RNAs versus delta HAZ score from DESeq2

transcript	baseMean	log2FoldChange	pvalue	padj	geneName
ENST00000345122.7_1	240.3212154	-4.780930929	4.80E-08	0.00034183	ARHGAP5
ENST00000376488.7_2	91.57992458	-4.185907335	3.73E-08	0.00034183	OTUD5
ENST00000381461.6_2	148.9208937	-4.505269214	5.15E-08	0.00034183	KIAA2026
ENST00000398263.6_1	420.8426705	-5.085179171	3.34E-08	0.00034183	TGOLN2
ENST00000452508.6_1	584.5910081	-6.757918139	2.03E-08	0.00034183	ATM
ENST00000380943.6_1	213.3141453	-5.480486639	1.76E-07	0.000972827	ERBIN
ENST00000231509.7_2	59.09814015	-3.653610934	3.58E-07	0.001696038	NR3C1
ENST00000517958.1_1	48.89576644	-3.639370889	4.17E-07	0.001731068	GALNT10
ENST00000570939.2_1	54.30149345	-3.864365355	5.28E-07	0.001946917	CREBBP
ENST00000353231.9_2	60.50676803	-3.651833949	6.09E-07	0.002021976	CLEC7A
ENST00000426229.1_2	295.894031	-3.576458258	7.20E-07	0.002173322	ADIPOR1
ENST00000354589.7_2	319.5230431	-3.20152824	1.20E-06	0.00305274	PLEC
ENST00000399765.5_1	70.80538696	-3.98551248	1.13E-06	0.00305274	BID
ENST00000620566.4_1	269.8800054	-4.471088908	1.82E-06	0.004323219	NUMA1
ENST00000311129.9_1	39.24125115	-3.812587412	2.55E-06	0.005047553	PPP2R1B
ENST00000331523.6_1	7797.459346	-4.1790863	2.57E-06	0.005047553	EEF1A1
ENST00000492354.1_1	161.9295903	-5.023909746	2.58E-06	0.005047553	SZRD1
ENST00000646076.1_1	78.37120897	-3.566997327	3.07E-06	0.00567048	CTCF
ENST00000532805.1_1	30.85593312	-3.570698242	3.38E-06	0.00590338	CYP2R1
ENST00000248248.7_1	93.72908338	-4.336984586	5.12E-06	0.006553807	MON1B
ENST00000263056.5_1	35.93462652	-2.870890418	3.96E-06	0.006553807	MAP3K8
ENST00000294339.3_1	100.3234483	3.987448272	4.39E-06	0.006553807	TAL1
ENST00000373330.1_1	46.98968351	-3.569444457	4.71E-06	0.006553807	ZMYM1
ENST00000404760.5_2	57.74032703	-2.790491092	4.49E-06	0.006553807	BRD1
ENST00000409663.7_1	370.2612459	-2.460426577	5.13E-06	0.006553807	KIAA0922
ENST00000503359.5_1	114.2088698	-4.106983267	4.98E-06	0.006553807	DCK
ENST00000465692.2_2	330.602008	-3.71340074	5.95E-06	0.007310127	RPS24
ENST00000496554.5_1	88.15083651	-4.331135018	7.52E-06	0.008792833	RPL7A
ENST00000647500.1_1	84.74280236	-4.221537964	7.68E-06	0.008792833	FRY
ENST00000344102.9_1	94.90750213	-3.89865107	8.97E-06	0.009633677	WARS
ENST00000636864.1_1	85.41681623	-4.644821575	9.00E-06	0.009633677	TBL1XR1
ENST00000333942.10_2	52.65849748	-3.836705919	1.04E-05	0.010178516	RHOT1
ENST00000448866.5_3	79.73965593	-4.257961559	1.04E-05	0.010178516	TBXAS1
ENST00000559717.5_1	53.61766316	-4.15284457	9.96E-06	0.010178516	MAN2A2
ENST00000376514.6_3	264.8851309	4.702292907	1.23E-05	0.010439561	LENG8
ENST00000392276.1_1	35.15260027	-3.608203176	1.18E-05	0.010439561	C19orf12
ENST00000539372.5_1	27.40304988	-3.135035297	1.21E-05	0.010439561	TNFRSF1A
ENST00000544180.6_1	73.98311442	-4.733039067	1.20E-05	0.010439561	PYGL
ENST00000644774.1_1	28.690728	-3.268702247	1.22E-05	0.010439561	ACTG1

ENST00000389044.8_2	38.16309052	-3.144288569	1.28E-05	0.010661927	TRIP12
ENST00000442341.5_1	236.9311209	-3.76944509	1.33E-05	0.010790242	RPL35A
ENST00000340480.8_1	23.21909816	-3.407794689	1.45E-05	0.011180002	HIPK1
ENST00000534600.5_1	63.16266779	-2.822438606	1.42E-05	0.011180002	API5
ENST00000340800.6_2	192.879238	2.959548237	1.61E-05	0.011222013	ACSL4
ENST00000374949.2_1	76.39239943	-4.088350647	1.72E-05	0.011222013	HLA-DQA1
ENST00000375383.7_2	51.45573368	-3.853028516	1.74E-05	0.011222013	KDM5C
ENST00000420239.6_1	61.32464955	-3.776368647	1.67E-05	0.011222013	CHD2
ENST00000436757.6_1	87.10924588	-4.87205118	1.68E-05	0.011222013	PITPNM1
ENST00000473364.1_1	17.13630335	-3.290314895	1.73E-05	0.011222013	MBNL3
ENST00000485963.5_1	22.25037521	-3.492532273	1.56E-05	0.011222013	PI4KA
ENST00000521308.5_1	66.74328662	-4.49196518	1.76E-05	0.011222013	CCDC69
ENST00000580306.6_1	49.49998733	-3.2631028	1.76E-05	0.011222013	ZNF18
ENST00000367697.7_1	21.63395157	-3.460304588	2.03E-05	0.012678849	HEBP2
ENST00000618666.4_1	210.9701953	-4.157497055	2.06E-05	0.012678849	CCNT1
ENST00000222254.12_1	33.98463757	-3.587065037	2.52E-05	0.01521029	PIK3R2
ENST00000261401.7_1	268.1011036	-3.394578158	2.67E-05	0.015820567	CORO1C
ENST00000346166.7_1	91.51818064	-2.965083075	2.76E-05	0.016085546	RNF6
ENST00000371884.6_1	95.26944461	-3.35579276	2.81E-05	0.016089943	TAL1
ENST00000356948.10_3	43.26420815	-3.593928582	3.06E-05	0.016911629	PTBP1
ENST00000579942.2_1	54.13895508	-4.44739775	3.03E-05	0.016911629	RASSF5
ENST00000242059.9_1	37.54804118	-3.18492712	3.37E-05	0.017050067	SCRN1
ENST00000298552.8_2	70.59979297	-3.876384415	3.25E-05	0.017050067	TSC1
ENST00000307714.12_1	44.11975252	-3.59660543	3.32E-05	0.017050067	KHDRBS1
ENST00000377658.8_1	52.20599014	-4.253902255	3.35E-05	0.017050067	KLHL21
ENST00000394986.5_1	260.1007438	-4.593695029	3.49E-05	0.017050067	SNCA
ENST00000493205.5_1	47.52477835	-3.956416925	3.45E-05	0.017050067	PTGES2
ENST00000557667.5_1	40.43130087	-2.590529794	3.35E-05	0.017050067	RBM23
ENST00000581544.5_1	89.03088003	-4.005567181	3.45E-05	0.017050067	EIF4A1
ENST00000414982.7_2	105.5194633	-4.149512689	3.57E-05	0.017184546	PNPLA6
ENST00000529957.5_1	14.36797428	-3.191360978	3.72E-05	0.017647045	GNPTG
ENST00000339121.9_1	21.06829223	-3.175704305	3.93E-05	0.017849266	ING3
ENST00000437149.6_1	15.19815864	-2.986595236	3.98E-05	0.017849266	ING4
ENST00000521604.6_1	131.5225703	-4.157302471	3.94E-05	0.017849266	TCEA1
ENST00000584583.1_1	73.60786517	-2.12427619	3.88E-05	0.017849266	RPL23
ENST00000368339.9_2	66.33482926	-4.437840904	4.04E-05	0.017876295	YY1AP1
ENST00000448187.5_1	63.88262704	-2.441930287	4.14E-05	0.018092688	NAP1L4
ENST00000537064.5_1	62.91573477	-3.858930973	4.59E-05	0.019807115	POLE
ENST00000331222.5_2	91.37392921	-2.952577433	4.76E-05	0.020280137	CLN8
ENST00000429490.5_1	59.17994133	-3.740168872	5.12E-05	0.021498057	SGMS1
ENST00000503857.5_1	43.99227558	-3.585574397	5.18E-05	0.021500705	YTHDC2
ENST00000326005.10_1	32.75212372	-3.606474511	5.27E-05	0.021607894	OAZ2
ENST00000579859.1_1	88.2793376	3.986735996	5.44E-05	0.022014379	RP5-1171I10.4

ENST00000394597.6_1	152.8793261	-2.241692616	5.81E-05	0.022818545	RFFL
ENST00000405731.7_1	27.46222744	-3.464770987	5.84E-05	0.022818545	ZDHHC4
ENST00000495776.5_1	33.32496045	-3.447515669	5.75E-05	0.022818545	OSBPL9
ENST00000470455.5_1	61.70386453	-3.537249469	6.06E-05	0.023365737	ATP6V1A
ENST00000566029.5_3	469.2759129	3.669433442	6.12E-05	0.023365737	CHD9
ENST00000581552.5_1	54.4333841	-2.404237932	6.31E-05	0.023794155	PIK3R5
ENST00000354250.6_1	32.79042737	-3.649263505	6.63E-05	0.024724975	NDUFV3
ENST00000268896.9_1	38.43698448	-3.911457314	6.92E-05	0.025514706	PCTP
ENST00000544778.6_1	287.2943936	3.177521574	7.02E-05	0.025592537	BPTF
ENST00000320254.5_1	38.81089182	-4.104069509	7.25E-05	0.026170916	LRRC37A
ENST00000371018.7_2	35.76796323	-4.314698386	8.54E-05	0.030155082	MIER1
ENST00000420959.6_1	212.236203	3.412293986	8.45E-05	0.030155082	CORO1C
ENST00000485846.5_1	65.57874102	3.785473614	8.63E-05	0.030164191	HBP1
ENST00000646577.1_1	11.73029641	-2.798903006	8.85E-05	0.030594112	DNAJC14
ENST00000347642.7_1	65.51460678	-4.375528865	9.08E-05	0.031064054	RNF14
ENST00000469867.1_1	23.78645593	-3.376557839	9.23E-05	0.031276445	FAM228B
ENST00000361510.7_2	117.9531591	3.373421764	9.33E-05	0.031301247	OPA1
ENST00000370441.8_2	34.91050995	-4.001972925	9.82E-05	0.031404394	IDS
ENST00000513750.5_1	26.41263534	-3.434275211	9.86E-05	0.031404394	ANKRD13D
ENST00000532074.5_1	19.73394138	-3.423530874	0.00010027	0.031404394	SSR2
ENST00000569637.6_1	12.36525884	-3.049383828	9.83E-05	0.031404394	NIP7
ENST00000578025.5_1	33.98845015	-3.846791782	0.000100241	0.031404394	RP5-1171I10.4
ENST00000602712.2_1	30.42685684	-3.447677606	9.94E-05	0.031404394	MARCH8
ENST00000612404.4_2	82.91530971	4.007263117	9.98E-05	0.031404394	SREK1
ENST00000342795.9_1	66.20865503	-1.809861022	0.000102196	0.031708569	CFLAR
ENST00000392437.6_2	145.2734651	-3.704984302	0.000103926	0.031946767	OPA1
ENST00000310544.8_1	32.56930163	-3.927190664	0.000108666	0.033097298	PHOSPHO1
ENST00000541960.5_1	74.04729649	-3.13351303	0.000111333	0.033601371	GABARAPL1
ENST00000302373.8_1	21.94358222	-3.374601431	0.000116362	0.034693178	MPHOSPH9
ENST00000361828.7_2	25.51404785	-3.301267025	0.000117041	0.034693178	OPA1
ENST00000216392.7_1	54.59057869	3.679857763	0.000121562	0.035191602	PYGL
ENST00000518683.5_1	80.82367734	-2.180423605	0.000121902	0.035191602	WWP1
ENST00000593064.5_2	23.50727579	-3.382049153	0.000121287	0.035191602	TCF3
ENST00000476562.5_1	84.10377017	-3.636104232	0.000126949	0.036026998	ELMSAN1
ENST00000579232.5_1	37.80519706	-3.304744992	0.000126966	0.036026998	THOC1
ENST00000645780.1_1	74.36322802	3.939774295	0.000129629	0.036470697	FRY
ENST00000520647.5_1	16.51983682	-2.937904271	0.00013573	0.037866375	GALNT10
ENST00000546017.5_1	36.97988026	-3.286105954	0.000143015	0.038917589	GABARAPL1
ENST00000549578.5_2	65.75813158	3.236248805	0.000141951	0.038917589	VPS29
ENST00000623724.3_1	203.5175149	4.321064301	0.000143008	0.038917589	IKZF3
ENST00000220531.7_1	243.0036549	-2.067007543	0.000146637	0.039259657	BLOC1S6
ENST00000440230.5_2	35.31375488	-2.024755174	0.000146515	0.039259657	LRRFIP2
ENST00000465632.5_1	28.90149921	-3.776982404	0.00015133	0.040191951	PGD

ENST00000245105.7_1	28.24006119	-3.603418709	0.000169185	0.044377887	SH3TC1
ENST00000330677.7_1	15.16898768	-2.614205017	0.000177201	0.044377887	NR2C1
ENST00000389061.9_1	30.62145547	3.319528176	0.000177784	0.044377887	SACM1L
ENST00000467081.1_1	26.49677163	-3.627310349	0.000177235	0.044377887	CCNL1
ENST00000478393.5_1	23.53499563	-3.463648983	0.000175001	0.044377887	ACTR3C
ENST00000479279.5_1	32.31760473	-2.8702744	0.000170414	0.044377887	WDR13
ENST00000494798.1_1	87.2658175	-2.845521517	0.000174976	0.044377887	CD96
ENST00000584944.5_1	17.11436386	-3.199987828	0.00017699	0.044377887	TRAF4
ENST00000370839.7_2	27.61134447	-4.329546539	0.000185101	0.045859473	MBNL3
ENST00000399976.6_1	40.87598538	-3.260402973	0.000188935	0.046120933	USP16
ENST00000418919.6_2	151.3718824	-3.732614599	0.00018835	0.046120933	GNS
ENST00000338972.8_2	26.18622852	-3.522213327	0.000192854	0.046395299	PAIP1
ENST00000534927.5_1	68.87845632	-2.988976793	0.000191496	0.046395299	NLRC5
ENST00000346027.9_1	31.36857287	-3.311870271	0.000200128	0.047798972	TLK2
ENST00000340023.6_1	23.48863001	-3.238353574	0.000206477	0.048615802	FPR2
ENST00000526060.5_4	58.4384499	3.237651432	0.000206358	0.048615802	SYVN1
ENST00000357496.6_1	87.30787825	-2.93503441	0.000211493	0.049446171	QRICH1
ENST00000613394.4_2	98.30801884	3.997096186	0.000214387	0.049772258	HPS1

Additional data table S1 (separate file)

Uchiyama_data_table_S1.xlsx: list of differential H3K4me3 peaks in one-year-old children with respect to delta HAZ score.

References

1. Kirkpatrick BD*, et al.* (2015) The "Performance of Rotavirus and Oral Polio Vaccines in Developing Countries" (PROVIDE) Study: Description of Methods of an Interventional Study Designed to Explore Complex Biologic Problems. *Am. J. Trop,.Med. Hyg.* 92:744-751.
2. Gilfillan GD*, et al.* (2012) Limitations and possibilities of low cell number ChIP-seq. *BMC Genomics* 13:645.
3. Adli M & Bernstein BE (2011) Whole-genome chromatin profiling from limited numbers of cells using nano-ChIP-seq. *Nat. Protoc.* 6:1656-1668.
4. Landt SG*, et al.* (2012) ChIP-seq guidelines and practices of the ENCODE and modENCODE consortia. *Genome Res.* 22:1813-1831.
5. O'Geen H, Echipare L, & Farnham PJ (2011) Using ChIP-seq technology to generate high-resolution profiles of histone modifications. *Methods Mol. Biol.* 791:265-286.
6. Vetesse-Dadey M*, et al.* (1996) Acetylation of Histone H4 Plays a Primary Role in Enhancing Transcription Factor Binding to Nucleosomal DNA In Vitro. *EMBO J.* 15:2508-2518.
7. Buonomo EL*, et al.* (2013) Role of interleukin 23 signaling in Clostridium difficile colitis. *J. Infect. Dis.* 208:917-920.

8. Flannigan KL, Geem D, Harusato A, & Denning TL (2015) Intestinal Antigen-Presenting Cells: Key Regulators of Immune Homeostasis and Inflammation. *Am. J. Pathol.* 185:1809-1819.
9. Yang J, Zhang L, Yu C, Yang X-F, & Wang H (2014) Monocyte and macrophage differentiation: circulation inflammatory monocyte as biomarker for inflammatory diseases. *Biomark. Res.* 2:1.
10. Khulan B*, et al.* (2012) Periconceptional maternal micronutrient supplementation is associated with widespread gender related changes in the epigenome: a study of a unique resource in the Gambia. *Hum. Mol. Genet.* 21:2086-2101.
11. Liu P & Hwang JTG (2007) Quick calculation for sample size while controlling false discovery rate with application to microarray analysis. *Bioinformatics* 23:739-746.
12. Orr M & Liu P (2009) Sample Size Estimation while Controlling False Discovery Rate for Microarray Experiments Using the ssize.fdr Package. *R Journal* 1:47-53.
13. Langmead B, Trapnell C, Pop M, & Salzberg SL (2009) Ultrafast and memory-efficient alignment of short DNA sequences to the human genome. *Genome Biol.* 10:R25.
14. Li H*, et al.* (2009) The Sequence alignment/map (SAM) format and SAMtools. . *Bioinformatics* 25:2078-2079.
15. Langmead B & Salzberg SL (2012) Fast gapped-read alignment with Bowtie 2. *Nat. Methods* 9:357-359.
16. Quinlan AR & Hall IM (2010) BEDTools: a flexible suite of utilities for comparing genomic features. *Bioinformatics* 26:841-842.
17. Kent WJ, Zweig AS, Barber G, Hinrichs AS, & Karolchik D (2010) BigWig and BigBed: enabling browsing of large distributed datasets. *Bioinformatics* 26:2204-2207.
18. Robinson JT*, et al.* (2011) Integrative Genomics Viewer. *Nat. Biotechnol.* 29:24-26.
19. Zhang Y*, et al.* (2008) Model-based analysis of ChIP-Seq (MACS). *Genome Biol.* 9:R137.
20. Feng J, Liu T, Qin B, Zhang Y, & Liu XS (2012) Identifying ChIP-seq enrichment using MACS. *Nat. Protoc.* 7:1728-1740.
21. Love MI, Huber W, & Anders S (2015) Moderated estimation of fold change and dispersion for RNA-seq data with DESeq2. *Genome Biol.* 15:550.
22. Dillies M-A*, et al.* (2013) A comprehensive evaluation of normalization methods for Illumina high-throughput RNA sequencing data analysis. *Briefings in Bioinformatics* 14:671-683.
23. Bailey T*, et al.* (2013) Practical Guidelines for the Comprehensive Analysis of ChIP-seq data. *PLoS Comp. Biol.* 9:e1003326.
24. Bonhoure N*, et al.* (2014) Quantifying ChIP-seq data: a spiking method providing an internal reference for sample-to-sample normalization. *Genome Res.* 24:1157-1168.
25. Flensburg C, Kinkel SA, Keniry A, Blewitt ME, & Oshlack A (2014) A comparison of control samples for ChIP-seq of histone modifications. *Front. Genet.* 25:xx.
26. Liang K & Keles S (2012) Normalization of ChIP-seq data with control. *BMC Bioinformatics* 13:199.
27. Taslim C, Huang K, Huang T, & Lin S (2012) Analyzing ChIP-seq Data: Preprocessing, Normalization, Differential Identification, and Binding Pattern Characterization. *Methods Mol. Biol.* 802:275-291.
28. Lovén J*, et al.* (2012) Revisiting Global Gene Expression Analysis. *Cell* 151:476-482.

29. Naylor C, *et al.* (2015) Environmental Enteropathy, Oral Vaccine Failure and Growth Faltering in Infants in Bangladesh. *EBioMedicine* 2:1759-1766.
30. McLean CY, *et al.* (2010) GREAT improves functional interpretation of cis-regulatory regions. *Nat. Biotechnol.* 28:495-501.
31. Huang da W, Sherman BT, & Lempicki RA (2009) Systematic and integrative analysis of large gene lists using DAVID bioinformatics resources. *Nat. Protoc.* 4:44-57.
32. Eden E, Navon R, Steinfeld I, Lipson D, & Yakhini Z (2009) GOrilla: A Tool For Discovery And Visualization of Enriched GO Terms in Ranked Gene Lists. *BMC Bioinformatics* 10:48.
33. Subramanian A, *et al.* (2005) Gene set enrichment analysis: A knowledge-based approach for interpreting genome-wide expression profiles. *PNAS* 102:15545–15550.
34. Draghici S, *et al.* (2007) A systems biology approach for pathway level analysis. *Genome Res.* 17:1537-1545.
35. Shen L, Shao N, Liu X, & Nestler EJ (2014) ngs.plot: Quick mining and visualization of next-generation sequencing data by integrating genomic databases. *BMC Genomics* 15:284.
36. Andersson R, *et al.* (2015) An atlas of active enhancers across human cell types and tissues. *Nature* 507:455-461.
37. Karolchik D, *et al.* (2004) The UCSC Table Browser data retrieval tool. *Nucleic Acids Res.* 32(Database issue):D493-D496.
38. Bailey TL & Elkan C (1994) Fitting a mixture model by expectation maximization to discover motifs in biopolymers. *Proceedings of the Second International Conference on Intelligent Systems for Molecular Biology.*, (AAAI Press, Meno Park, California), pp 28-36.
39. Wood ARea (2014) Defining the role of common variation in the genomic and biological architecture of adult human height. *Nat. Genetics* 46:1173-1186.
40. Mentch SJ, *et al.* (2015) Histone Methylation Dynamics and Gene Regulation Occur through the Sensing of One-Carbon Metabolism. *Cell Metab.* 22:861–873.
41. Kim D, Langmead B, & Salzberg SL (2015) HISAT: a fast spliced aligner with low memory requirements. *Nat. Methods* 12:357–360.
42. Pertea M, *et al.* (2015) StringTie enables improved reconstruction of a transcriptome from RNA-seq reads. *Nat. Biotechnol.* 33:290-295.
43. Lenth RV (2006) Java Applets for Power and Sample Size).
44. Hulsen T, de Vlieg J, & Alkema W (2008) BioVenn – a web application for the comparison and visualization of biological lists using area-proportional Venn diagrams. *BMC Genomics* 9:488.
45. Consortium TEP (2012) An integrated encyclopedia of DNA elements in the human genome. *Nature* 489:57-74.
46. Zhu J, *et al.* (2013) Genome-wide Chromatin State Transitions Associated with Developmental and Environmental Cues. *Cell* 152:642-654.
47. Calo E & Wysocka J (2013) Modification of Enhancer Chromatin: What, How, and Why? *Mol. Cell* 49:825-837.
48. Brunet A, *et al.* (1999) Akt promotes cell survival by phosphorylating and inhibiting a Forkhead transcription factor. *Cell* 96:857-868.
49. Lillis AP, *et al.* (2015) LDL Receptor-Related Protein-1 (LRP1) Regulates Cholesterol Accumulation in Macrophages. *PLoS ONE* 11:e0147457.

50. Lillis AP, Van Duyn LB, Murphy-Ullrich JE, & Strickland DK (2008) LDL Receptor-Related Protein 1: Unique Tissue-Specific Functions Revealed by Selective Gene Knockout Studies. *Physiol. Rev.* 88:887-918.
